# A supraparticle-based biomimetic cascade catalyst for continuous flow reaction

**DOI:** 10.1038/s41467-022-33756-1

**Published:** 2022-10-08

**Authors:** Xiaomiao Guo, Nan Xue, Ming Zhang, Rammile Ettelaie, Hengquan Yang

**Affiliations:** 1 School of Chemistry and Chemical Engineering, Shanxi University, 030006 Taiyuan, China; 2 Food Colloids Group, School of Food Science and Nutrition, University of Leeds, Leeds, LS2 9JT UK; 3 Key Laboratory of Chemical Biology and Molecular Engineering of Ministry of Education, Shanxi University, 030006 Taiyuan, China

**Keywords:** Heterogeneous catalysis, Catalyst synthesis, Chemical engineering

## Abstract

Robust millimeter-sized spherical particles with controlled compositions and microstructures hold promises of important practical applications especially in relation to continuous flow cascade catalysis. However, the efficient fabrication methods for producing such particles remain scare. Here, we demonstrate a liquid marble approach to fabricate robust mm-sized porous supraparticles (SPs) through the bottom-up assembly of silica nanoparticles in the presence of strength additive or surface interactions, without the need for the specific liquid-repellent surfaces used by the existing methods. As the proof of the concept, our method was exemplified by fabricating biomimetic cascade catalysts through assembly of two types of well-defined catalytically active nanoparticles. The obtained SP-based cascade catalysts work well in industrially preferred fixed-bed reactors, exhibiting excellent catalysis efficiency, controlled reaction kinetics, high enantioselectivity (99% ee) and outstanding stability (200~500 h) in the cascades of ketone hydrogenation-kinetic resolution and amine racemization-kinetic resolution. The excellent catalytic performances are attributed to the structural features, reconciling close proximity of different catalytic sites and their sufficient spatial isolation.

## Abstract (web summary)

Robust millimeter-sized spherical particles with controlled compositions and microstructures hold promises of important practical applications. Here the authors develop a liquid marble method to facilely fabricate robust millimeter-sized supraparticles with controlled microstructures through the bottom-up assembly of silica nanoparticles.

## Introduction

Cascade catalysis in a continuous flow fashion is a step-change route to upgrade the stepwise fine chemical synthesis due to the collective advantages of continuous flow and cascade reactions, such as enhanced reaction efficiency, reduced energy consumption, avoidance of intermediate separation and beneficial shifting reaction equilibrium^1–5^. One central issue in successful application of this concept in industry relates to the formation of the catalysts, which should be solid particles with sizes large enough to be in millimeter scale, withstand high mechanical strength, and mange to integrate two or more distinct catalytic sites in close proximity^6,7^. In pursuit of this goal, various methods or techniques have been explored including physical mixing of two or more different catalyst particles in a fixed-bed reactor^8–10^, multi-layering of different catalyst particles in a column reactor^11^, and two-stage reactions linked in series^12,13^. Despite successful combination of different catalysts together, these methods are still not capable of controlling individual catalytic steps to occur in nanoscale proximity because different catalyst sites are physically separated in different large-sized particles. Co-localization of different catalytic sites in different regions of a single particle can shorten the distances between different catalytic sites^14–16^. This strategy, however, is currently limited to fabrication of nanometer-sized materials, and is incapable of selective-region positioning of different catalytic sites onto a single millimeter-sized particle which is industrially required^17,18^. We envisage that if two or more kinds of well-defined catalytically active nanoparticles (NPs), for example metal-supported or enzyme-immobilized silica NPs, are integrated into the structure of robust millimeter-sized particles in a manner similar to cells that can assemble multiple different enzymes within a cellular compartment, the concept of continuous flow cascade catalysis will be significantly advanced toward the level of practical applications.

Supraparticles (SPs) are an emerging concept on bottom-up assembly of primary NPs to form larger objects. Such a technique holds the potential for preparing large-sized microspheres with well-defined structures on nanoscales^19–26^. Beyond this, SPs can be endowed with additional or collective properties, which are otherwise unattainable in dispersions of individual NPs alone^27^. Spray drying and emulsification methods were extensively developed to fabricate SPs using small particles as building blocks^28–31^. However, the spray drying method hardly generates mm-scale microspheres because of the deformation of droplet and generation of cracks caused by rapid liquid evaporation at high temperatures. Similarly, the emulsion method fails to fabricate mm-sized SPs since large emulsion droplets are not stable enough to sever as suitable template for the assembly of NPs. Another emerging method is the evaporation of NPs-containing liquid drops on superhydrophobic/superamphiphobic surfaces^19–22^, or drying of NPs-containing liquid drops on hydrophobic surfaces with the aid of oil as lubricant^23^. Despite being very appealing, these existing methods require either specific surfaces that are very repellent to the liquid involved, or liquids that have very specific compositions. The microfluidic method was also explored to manufacture SPs, yet has been unable to obtain required mm-sized SPs^27^. Critically, SPs generated with these methods still suffer from low mechanical strength because of lack of strong adhesion between the NPs^27,32^. As such, effective, bottom-up methods to fabricate robust SPs and further SP-based cascade catalysts especially in desired mm-scaled sizes remain a scientific and technological challenge.

In this work, aiming at addressing the above fundamental and practical issues, we develop a liquid marble method to fabricate a SP-based biomimetic cascade catalyst and explore its applications in continuous flow cascade reactions. We demonstrate how to control the morphology and mechanical strength of SPs, and further identify the key factors that impact their properties. Rather impressively, the mechanical strength of the obtained SPs reaches values as high as 18.5 N, which can even be comparable to that of ordinary particles obtained through calcination. Furthermore, a SP-based catalyst consisting of both Pd-supported and enzyme-immobilized silica NPs was fabricated to exemplify the use of this liquid marble protocol. The SP-based catalyst was shown to reconcile high activity and long-term stability in industrially preferred fixed-bed reactors, which is demonstrated by two distinct cascade reactions. The proximity effect and isolation effect arising from SPs were found to be crucial in achieving the excellent activity and long-term stability.

## Results

### Supraparticle design and preparation

Liquid marble was chosen as micro-compartments within which nanoparticle building blocks are assembled, because of its unique properties. Liquid marble is a well-known interfacial phenomenon, where liquid was compartmentalized into 0.01–10 *μ*L drops whose surfaces are covered by a layer of hydrophobic NPs^33–35^. Because the particles at surfaces are trapped in a deep energy well arising from large adsorption energy^36^, liquid marbles have good mechanical stability against deformation during transport and are non-stick toward solid substrate surface with which they are in contact. Another noteworthy advantage of liquid marbles is their ability to retard evaporation of the inner liquid because of the presence of a hydrophobic layer around the drops^37,38^, supported by our evaporation experiments (Supplementary Fig. 1). These advantages are helpful to control the evaporation-induced assembly process. Spongy silica particles modified with octyltrimethoxysilane were used to form a hydrophobic layer around drops (Supplementary Fig. 2)^39^. Mesoporous silica nanospheres (MSNs) with diameters of *ca*. 100 nm were utilized as building blocks considering that their nanopores are not only useful for the loading of catalytic sites but also advantageous to create highly porous SPs (Supplementary Fig. 3). Tetraethylenepentamine (TEPA) was harnessed as strength additive to reinforce SPs because hydrogen-bonds are expected to be formed between TEPA and silanols on the surfaces of MSNs^40,41^.

**Fig. 1:**
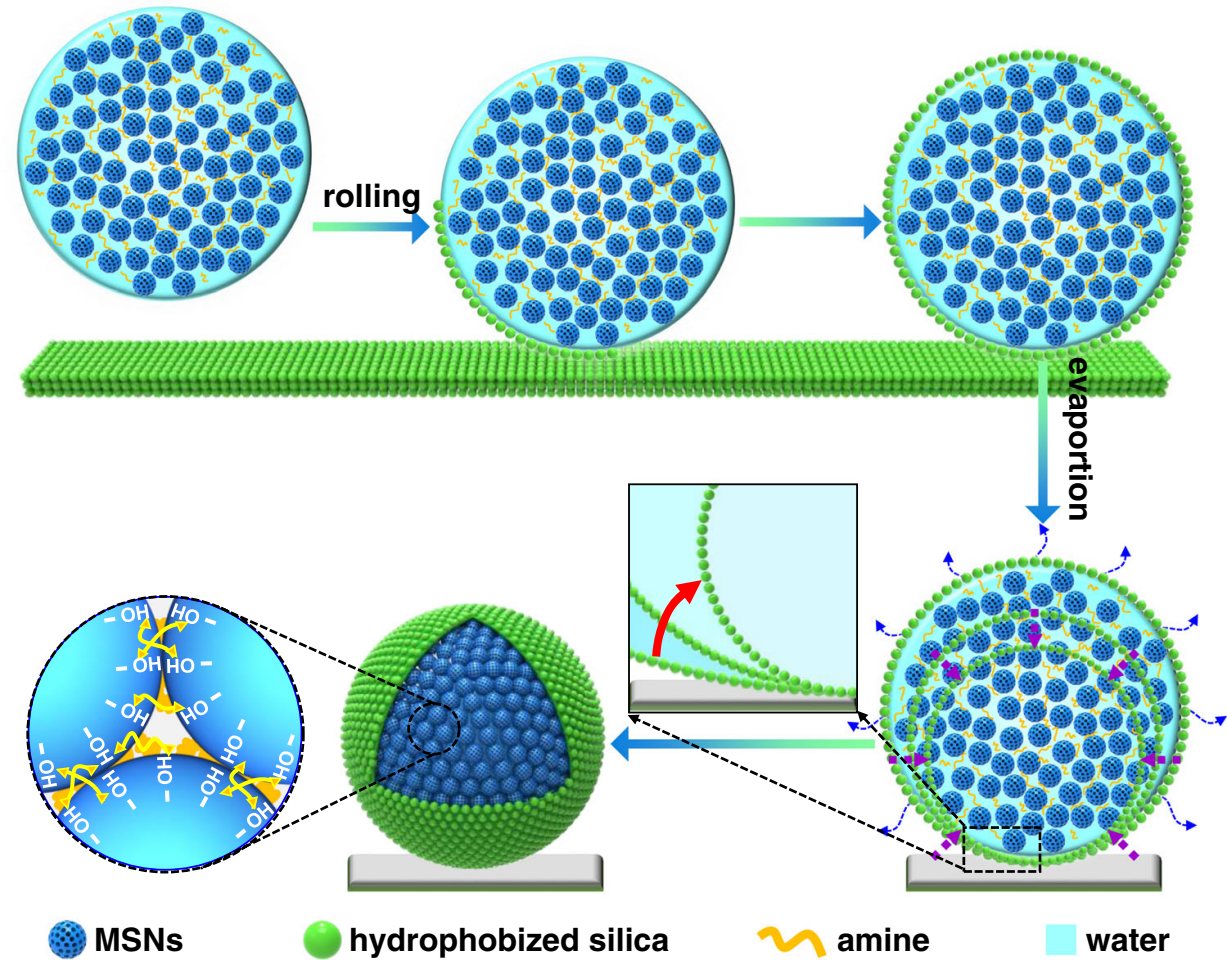
Schematic illustration of the preparation of SPs. Droplets of TEPA solution containing MSNs are dropped onto a bed made of hydrophobized spongy silica powders, followed by evaporating of water within liquid marbles under ambient conditions.

The SP fabrication process is schematically depicted in Fig. 1, consisting of two steps: preparation of liquid marbles and evaporation-induced assembly. An aqueous suspension containing 4 wt% TEPA and 10 wt% MSNs (with respect to the total mass of water, TEPA and MSNs, otherwise mentioned) were dropped onto a bed made of hydrophobized spongy silica powders using a syringe pump, followed by rolling on this bed. Due to the gas/liquid interfacial adsorption, the hydrophobic silica particles were picked up from the bed, forming a hydrophobic shell around the water drops. The generated liquid marbles were transferred onto a solid substrate (for example, paper as a very cheap solid substrate). Then, evaporation of water within liquid marbles under ambient conditions causes their surface to shrink toward to the center and concomitant assembly of MSNs within the confined space of liquid marbles in the aid of TEPA. The key role of liquid marbles in evolution of uniform spherical SPs is highlighted by a control experiment, where bare drops (without adsorption of hydrophobic silica particles on the surfaces) led to a mooncake-like shape with some cracks (Supplementary Fig. 4). Our liquid marble strategy avoids pinning of the three-phase contact line arising from the direct contact of the liquid with the substrate surfaces, thereby ensuring the hydrophobic silica particles adsorbed at the interfaces gradually move toward the center as the droplets shrinks during the evaporation. Another key factor is slow evaporation of water out of the liquid marble, which can avoid coffee-ring effects to some extent since the slowed down evaporation is helpful for reducing the mobility of MSNs to the interfaces^42–44^. This inference is supported by the rapid evaporation experiments where detrimental impacts of not having slow evaporation are demonstrated (Supplementary Fig. 5). These two factors contribute to an isotropic shrinkage of droplet, eventually leading to a uniform spherical morphology. Overall, our method holds promises for large-scale fabrication because of its inherent operational simplicity.

### Structure and mechanical strength of SPs

**Fig. 2:**
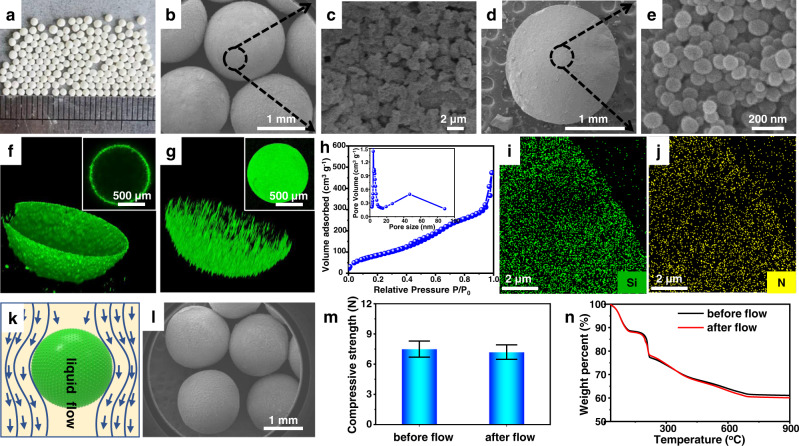
Structural characterization of SPs. **a** Appearance of SPs. **b** SEM image of SPs. **c** SEM image showing the surface of SPs. **d** SEM image showing the interior structure of SPs, after being cut deliberately. **e** Magnified SEM image showing the interior structure of SPs. **f** Fluorescence confocal microscopy image of SPs with hydrophobized spongy silica powders labelled by FITC-I. **g** Fluorescence confocal microscopy image of SPs with MSNs labelled by FITC-I. **h** N_2_ adsorption-desorption isotherms of SPs (the inset is the pore size distribution plot). **i** Si elemental mapping of the interior of SPs. **j** N elemental mapping of the interior of SPs. **k** Schematic illustration of the SPs tested in a fixed-bed reactor. **l** SEM image of the SPs after the test. **m** Mechanical strength of SPs before and after the test. Error bars indicate the standard error from 20 samples averages. **n** TGA profiles of SPs before and after the test.

The morphology and structures of the obtained SPs were systematically characterized. For example, SPs prepared with 4 wt% TEPA and 10 wt% MSNs take the form of discrete microspheres with an average diameter of 1.5 mm, as seen from scanning electron microscopy (SEM) images in Fig. 2a, b. The surfaces of SPs are covered with spongy silica particles (Fig. 2c and Supplementary Fig. 6), while its interior is filled with numerous MSNs (Fig. 2d, e). These locations are consistent with the fluorescent observations (Fig. 2f, g). When spongy silica particles were labelled with a fluorescent reagent, isothiocyanate isomer, a green fluorescent circle around SPs was clearly observed. In contrast, when MSNs were labelled with this fluorescent reagent, fluorescent signals were present throughout the interior of SPs. The strong interfacial adsorption of spongy silica particles to the surfaces of liquid marbles ensures that these particles will remain at the interface. In contrast, confined to the body of liquid marbles, the assembly of MSNs is guaranteed to stay solely within the interior. N_2_ sorption analysis reveals a bimodal porosity nature of SPs, as evidenced by two moderately steep adsorption steps at relative pressures (P/P_0_) of 0.4–0.7 and >0.95 (Fig. 2h). *Ca* 4 nm pore is related to the pores within MSNs, while 47 nm pores are attributed to the interspaces formed among the packed MSNs. The BET specific surface area and pore volume were determined to be 347 m^2^ g^−1^ and 0.73 cm^3^ g^−1^, respectively. There is a remarkable decrease in the specific surface area in comparison to the primary MSNs, resulting from the introduction of TEPA into pores. In order to assay the permeability of SPs toward external molecules, a fluorescent reagent (Rhodamine B), was used to visualize permeation process (Supplementary Fig. 7). Rhodamine B was observed to pass through the edge of SPs, as evident by a fluorescent circle at the beginning. This was followed by a progressive increase in the fluorescent intensity within the interior region, attributed to the continuous diffusion of more fluorescent molecules into SPs. The intensity eventually levels off after about 50 s as the inside of SPs become saturated with fluorescent molecules. This observation reflects the excellent accessibility the interior of SPs toward ingress of external molecules, which arises from their highly porous architecture. Elemental mappings of SPs reveal that Si and N elements are uniformly distributed throughout the whole body of SPs (Fig. 2i, j), indicating both MSNs and TEPA are homogeneously organized at a microscale level. Moreover, at fixed TEPA and MSN concentrations, larger-sized liquid marbles gave rise to larger SPs because of the compartmentalization effect of liquid marbles (Supplementary Fig. 8). It is noteworthy to mention that the liquid marble strategy allows us to achieve SPs as large as 2.1 mm. Such a size is close to the limitation of forming spherical drops, which will deform upon further increasing the drop size because the capillary force no longer favorably counterbalances the gravity^22^.

The mechanical strength of SPs which is another crux for practical applications, was evaluated. The produced SPs could withstand a mechanical strength of 7.5 N (for a single particle), which was estimated by compressing tests. More impressively, the mechanical strength of SPs can even be favorably comparable to that of the SPs after calcination at 550 or 800 °C (Supplementary Fig. 9). Therefore, our calcination-free protocol provides the possibility for the bottom-up assembly of catalytically active NPs into large-sized particles, even when the NPs may be sensitive to high temperature treatment. Such a high mechanical strength encouraged us to test the stability of SPs under industrially simulated conditions. Being millimeter-sized spheres, the obtained SPs could be directly packed in a fixed-bed reactor. After 48 h of treatment with flowing *n*-octane (Fig. 2k, 4 MPa, 6 mL h^−1^), SPs still retained their original spherical shape and structural integrity, and no fractures on their surfaces were observed, as evident from the SEM images (Fig. 2l). There is no decrease in the mechanical strength after the treatment either (Fig. 2m). Moreover, Fig. 2n displays the TGA results of SPs before and after the treatment. Their comparison indicates that the strength additive was completely retained within SPs. It is the same with the use of other solvents such as toluene, ethyl acetate but with exception of the strongly polar dimethyl sulfoxide (Supplementary Fig. 10, in the case of dimethyl sulfoxide, a loss was observed but only at a modest 4.6% weight loss), suggesting a general inference that TEPA molecules are firmly linked with MSNs. Another test was conducted with a fluidized bed reactor. After 48 h of fluidization treatment (N_2_ at a superficial velocity ca. 0.3 m s^–1^), the spherical morphology was virtually unaltered (Supplementary Fig. 11). It is the same case with the SPs prepared with 20 wt% MSNs (Supplementary Fig. 12). These results clearly demonstrate the SPs prepared in the above manner have sufficiently high mechanical strength to render them amenable to utilization in practical applications.

### Key parameters impacting the mechanical strength

**Fig. 3:**
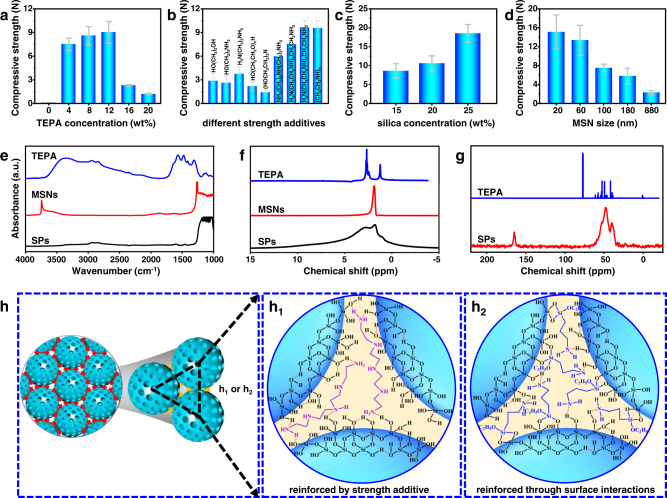
Key factors impacting of the mechanical strength of SPs. **a** Mechanical strength of SPs prepared with different concentrations of TEPA. **b** Mechanical strength for SPs prepared with different strength additives. **c** Mechanical strength of SPs prepared with different concentrations of MSNs. **d** Mechanical strength for SPs prepared with different sized MSNs. Error bars indicate the standard error from 20 samples averages in figure (**a**–**d)**. **e** FT-IR spectra of TEPA, MSNs and SPs. **f** ^1^H NMR spectrum of TEPA and ^1^H solid-state NMR spectra of MSNs and SPs. **g** ^13^C solid-state NMR spectra of TEPA and MSNs. **h** Proposed mechanism for reinforcement in SPs. **h**_**1**_ Reinforcement by strength additive. **h**_**2**_ Reinforcement through surface interactions.

With the above interesting results, we next identified the key parameters which impact the mechanical strength of SPs. To this end, we independently varied the TEPA concentration, strength additive, MSN concentration, and MSN size. As mentioned above, the particles prepared in the absence of TEPA exhibits a pan-like shape, whose mechanical strength is below the detection limit (<0.1 N, Supplementary Fig. 13). The significantly enhanced mechanical strength and the desired spherical morphology manifest the important role of TEPA. When the TEPA amount was increased up to 4 wt% TEPA, then to 8 wt%, 12 wt% and 16 wt%, while keeping the MSN concentration constant at 10 wt% (Supplementary Fig. 13), we observed formation of uniform spherical SPs, with mechanical strength increasing at first, peaking at 12 wt% TEPA (9 N, Fig. 3a). However, further increase of TEPA to 16% caused a decrease in mechanical strength because of excessive liquid filling among MSNs. The presence of TEPA results in a significant increase in the viscosity of liquids (Supplementary Fig. 14). Such a viscosity enhancement will probably suppress capillary flows and thereof coffee-ring effect that are unfavorable to form spherical shape^45^. At the same time, we also found that the mechanical strength of SPs was also governed by the structure and functionality of the strength additives (Fig. 3b and Supplementary Fig. 15). When diethylenetriamine (DETA) or triethanolamine was used, the mechanical strength was <6 N. As the number of N−H bond in the additive molecules increases, so does the mechanical strength. For the case of multi-amine polymer (PEI), the mechanical strength reached values as high as 9.5 N. We found that the N−H bond much better in reinforcing the mechanical strength, as compared to the O−H bond. When ethanolamine and polyethylene glycol were used, the mechanical strength was only 2.6 and 2.2 N, respectively. These values are much smaller than those prepared with the corresponding amines. Moreover, the amine family of strength additives were found to be better than chitosan and epoxy that are often used as agents for shaping and enhanced stiffness (Supplementary Fig. 16)^46,47^. Furthermore, we observed that the mechanical strength of SPs is strongly dependent on the MSN concentration. In the absence of MSNs, it is hard to obtain spherical SPs (Supplementary Fig. 17). The mechanical strength increases monotonously with the MSN concentration increasing from 10 wt% to 25 wt% (Fig. 3c). Notably, the SPs prepared with 25 wt% MSNs could withstand a compressive strength of 18.5 N. However, when more MSNs were applied, it is harder to prepare liquid marbles because of difficulty in shaping the suspension to spherical drops. At the same time, the MSN concentration can influence the size of SPs (Supplementary Fig. 18). Under the same conditions, a higher MSN concentration led to bigger SPs. Interestingly, we found that the MSN size also dictates the mechanical strength (Supplementary Figs. 19 and 20), with the mechanical strength drastically increasing when MSN particles were made smaller (Fig. 3d). For example, a mechanical strength as high as 15 N was achieved for the case involving 10 wt% MSNs. The porosity of SPs also changes with altering the above parameters, indicating a good tunability of the porosity of fabricated SPs (Supplementary Figs. 21–23).

To clarify the reason for the high mechanical robustness, we employed Fourier transform infrared spectroscopy (FT-IR) and solid-state NMR spectroscopy, in order to study the interactions between MSNs and amines. As seen from the FT-IR spectrum of MSNs, a peak for the stretching vibrations of O−H on the silica surfaces (3740 cm^−1^) was clearly observed (Fig. 3e). In contrast, this peak completely disappeared for SPs, which may be due to the formation of hydrogen-bonding interactions between TEPA and Si−OH on the MSN surfaces. The FT-IR results are supported by ^1^H MAS NMR results (Fig. 3f). For TEPA in liquid, a peak at 1.2 ppm was clearly detected, which can be assigned to H (N*H*_*2*_ or N*H*); for MSNs, there is a peak at 1.8 ppm, which corresponds to H (SiO*H*). However, for SPs, a very broad peak in the range 4–8 ppm was visible, which is not only different from that for TEPA and MSNs on their own, but also is not a simple combination thereof. These changes suggest that there exist strong interactions between TEPA and MSN surfaces. Figure 3g shows ^13^C NMR spectrum of TEPA in liquid and the ^13^C solid-state NMR spectrum of SPs. Comparison of these two spectra reveals that the C signals corresponding to TEPA were found in SPs; but more significantly these peaks are apparently widened in comparison to the liquid state TEPA, implying that the mobility of TEPA molecules in SPs are largely restricted by the existing interactions.

Based on the above results, we can elaborate the enhanced mechanical strength of SPs according to the equation^48^:

1 $$\sigma=\frac{{{{{{\rm{nF}}}}}}(1-{{{{{\rm{\theta }}}}}})}{{{{{{\rm{\pi }}}}}}{{{{{{\rm{d}}}}}}}^{2}}$$

where *σ* is the mechanical strength, *n* is the contact point, *F* is the binding force, *d* is the particle diameter and *θ* is the porosity. According to this equation, there are three key parameters: *F*, *n* and *θ*, which jointly govern the mechanical strength. The hydrogen-bonding interactions between amines and silanols on MSN surfaces provide a binding force (*F*) for holding MSNs together (Fig. 3h, h1). The stronger the interactions, the higher the mechanical strength. The appropriate increase in the amount of strength additive or altering the type of strength additives can improve the mechanical strength, which are in full supported by our above experiments. MSNs can provide numerous pillars within SPs at the places of contact (*n*). The smaller the building blocks, the more the contact points (*n*) per unit volume and the smaller the porosity (*θ*), which can be imaged according to Fig. 3h. As a result, the mechanical strength increases upon decreasing the size of MSNs, which also agrees with the above experimental observations. Moreover, this method is applicable to assembly of other NPs, such as TiO_2_ or microporous H-beta zeolite (Supplementary Fig. 24). More interestingly, the principle of enhanced strength via interparticle interactions can be extended to surface-modified systems, where SPs are reinforced through surface interactions between grafted amines and silanols (Fig. 3h2). For example, using 3-aminopropyltriethoxysilane- or trimethoxysilyl propyl DETA-grafted MSNs or enzyme-immobilized MSNs as building blocks, the resultant SPs can withstand a mechanical strength of 3.2 N without any extra additives (Supplementary Fig. 25).

### Design and characterization of SP-based cascade catalyst

**Fig. 4:**
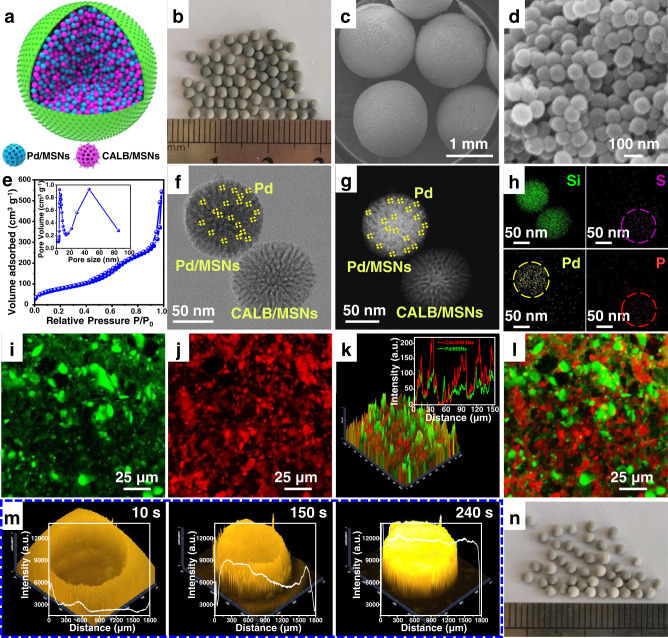
Characterization of the SP-based cascade catalyst. **a** Structural illustration of the microstructures of Pd-CALB/SPs. **b** Appearance of Pd-CALB/SPs. **c** SEM image showing Pd-CALB/SPs. **d** SEM image showing the interior structure of Pd-CALB/SPs. **e** N_2_ adsorption-desorption isotherms of Pd-CALB/SPs (the inset is the pore size distribution plot). **f** High-resolution TEM image showing the building blocks within Pd-CALB/SPs. **g** High-resolution TEM image showing the building blocks within Pd-CALB/SPs. **h** Elemental mapping of the interior of Pd-CALB/SPs. **i** Fluorescence confocal microscopy image of Pd-CALB/SPs with Pd/MSNs labelled by FITC-I. **j** Fluorescence confocal microscopy image of Pd-CALB/SPs with CALB/MSNs labelled by Rhodamine B. **k** 2.5D confocal laser scanning microscopy image of Pd-CALB/SPs with Pd-MSNs and CALB-MSNs labelled by FITC-I and Rhodamine B, respectively (the inset is fluorescence intensity profiles showing the distribution of Pd/MSNs and CALB/MSNs within the Pd-CALB/SPs). **l** Fluorescence confocal microscopy image of the interior of Pd-CALB/SPs with Pd-MSNs and CALB-MSNs labelled by FITC-I and Rhodamine B, respectively. **m** Time-dependent fluorescence intensity for the transport of Rhodamine B within a Pd-CALB/SPs particle. **n** Appearance of Pd-CALB/SPs after the continuous flow treatment.

Next, we transferred our SP concept to the design of a true cascade catalyst. Pd-supported MSNs and lipase CALB-immobilized MSNs were chosen as building blocks because they represent a typical combination of chemo- and biocatalysts, having important implications for many cascade reactions^49^, for example sequential ketone hydrogenation−alcohol kinetic resolution^50–53^, and sequential amine kinetic resolution−amine racemization^54–57^. Metal Pd NPs with an average size of 1 nm were supported on amine-modified MSNs (Pd/MSNs, Supplementary Fig. 26); CALB was immobilized on octyl-modified MSNs (CALB/MSNs, Supplementary Fig. 26) through physical adsorption. The prepared SP-based cascade catalyst is denoted as Pd-CALB/SPs, whose structure is schematically illustrated in Fig. 4a.

The obtained Pd-CALB/SPs exhibit discrete spherical shape with diameter of 1.5 mm and their interior consists of numerous MSNs (Fig. 4b–d). Like SPs, Pd-CALB/SPs exhibited a bimodal pore distribution (Fig. 4e). As TEM image revealed (Supplementary Fig. 27a), most of Pd NPs were distributed in the nanopores (0.63 wt% Pd, Supplementary Table 1). To check the location of CALB in Pd-CALB/SPs, we labelled CALB with Au clusters for TEM observation (Supplementary Fig. 27b). Au clusters were found to be uniformly present inside the nanopores of MSNs, mirroring a fact that CALB molecules were distributed inside the nanopores too (CALB loading, 46.5 mg g^−1^). N_2_ sorption analysis further confirms that both Pd NPs and CALB entered the pores of MSNs because their pore size decreased from 4.5 to 4.1, and from 4.7 to 4.2 nm, respectively (4.5 nm for amine-modified MSNs, 4.7 nm for octyl-modified MSNs, Supplementary Figs. 28 and 29). Meanwhile, TEM images of Pd-CALB/SPs in Fig. 4f, g show that Pd/MSNs and CALB/MSNs are distributed within the SP but are in close proximity, which is supported by the elemental mapping results in Fig. 4h. Furthermore, we separately labelled Pd-supported MSNs with FITC-I and CALB-immobilized MSNs with Rhodamine B during the preparation of SP-based catalyst (See Supplementary Experiments). Green fluorescent signals were found to be present throughout a SP entity (Fig. 4i), whilst red fluorescent signals also appeared everywhere in the body of SPs (Fig. 4j). When these two types of NPs were simultaneously labelled fluorescently, both red and green signals are visible together, also affirming a mixed homogeneous distribution of these two types of NPs within SPs (Fig. 4k, l, Supplementary Fig. 30). Moreover, Pd-CALB/SPs exhibits a good permeability to external molecules. Within the first 10 s, fluorescence appears at the SP periphery, and after 240 s the concave-shaped fluorescent pattern gradually changes to a bulge-shaped pattern, implying that fluorescent molecules reached the center of SPs (Fig. 4m). Such a good permeability is greatly beneficial to catalysis reactions occurring inside the SP-based catalyst.

### Cascade of hydrogenation and kinetic resolution over Pd-CALB/SPs

**Fig. 5:**
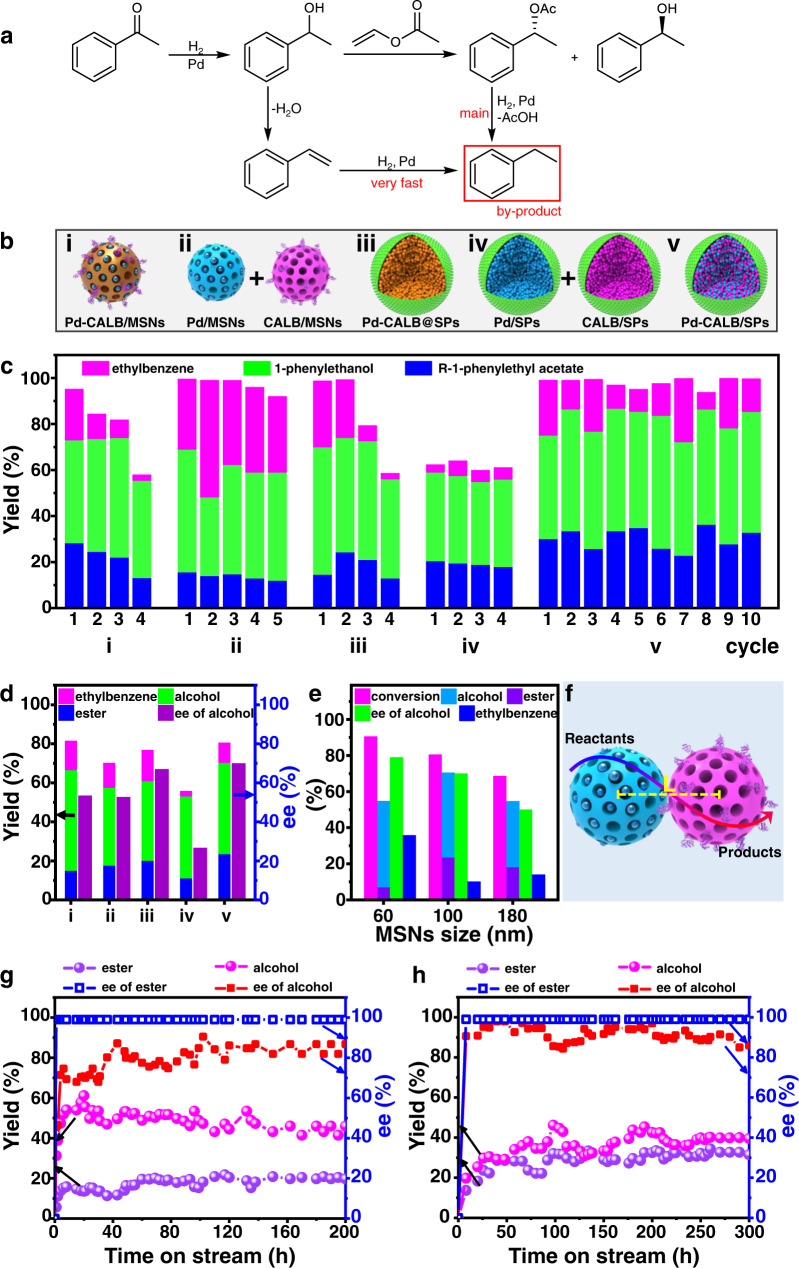
Results of the cascade of hydrogenation and kinetic resolution over different catalysts. **a** Reaction network of the cascade reaction. **b** Schematic illustration of the structures of five analogous catalysts. **c** Recyclability of these five catalysts, reaction conditions: 0.2 mmol acetophenone, 0.8 mmol vinyl acetate, 2.0 mL *n*-octane, 50 °C, 2 MPa H_2_, 6 h, S/C = 56:1 (substrate to Pd), 3.52–3.55 *μ*mol Pd and 1.40–1.42 mg CALB applied for all the batch reactions. **d** Results of the cascade reaction over different catalysts, reaction conditions are the same as in (**c**) except reaction time (4 h). **e** Results of the cascade reactions over SPs prepared with differently sized MSNs, reaction conditions are the same as in (**d**). **f** Schematic illustration for proximity effects. **g** Continuous flow cascade reaction using acetophenone as substrate, flow rate from initial 3 mL h^−1^ to 0.9 mL h^−1^ at the end. **h** Continuous flow cascade reaction using 4′-methylacetophenone as substrate, flow rate from initial 2 mL h^−1^ to 0.6 mL h^−1^ at the end. Reaction conditions: 0.1 M substrate and vinyl acetate (0.4 M) in *n*-octane, 50 °C, 2 MPa H_2_ (10 mL min^−1^).

The cascade of ketone hydrogenation to racemic alcohols and kinetic resolution of racemic alcohols is a potential method for one-pot synthesis of two chiral products: alcohol and ester^50–53^. In this cascade reaction, hydrogenation of acetophenone over Pd NPs gives racemic 1-phenylethanol and then one enantiomer of 1-phenylethanol is acylated to chiral phenylethyl acetate leaving the other enantiomer intact (Fig. 5a). Despite extensive effort, the yields of chiral *R*−1-phenylethyl acetate and 1-phenylethanol are not yet satisfactory because side-reactions such as debenzylation of *R*−1-phenylethyl acetate or 1-phenylethanol to styrene, and hydrogenation of styrene to ethylbenzene, can take place^50,51^.

To benchmark our SP-based catalyst, we first compared Pd-CALB/SPs with its analogues in batch reactions (50 °C, 2.0 MPa H_2_). The initial choice for using batch reactions was made since some of the analogous catalysts are in form of NPs. As such, it is not feasible to directly test them in fixed-bed reactors. The analogues included a catalyst with Pd NPs and CALB co-localized on a single MSN particle (Pd-CALB/MSNs), a physical mixture of Pd/MSNs and CALB/MSNs, a physical mixture of Pd/SPs and CALB/SPs (i.e., separate assembly of Pd/MSNs or CALB/MSNs into SPs), and Pd-CALB@SPs which involves assembly of Pd-CALB/MSNs into SPs (i.e., where pores of each MSN particle simultaneously contained both Pd and CALB). A schematic illustration of all different attempted analogues is presented in Fig. 5b (from i to v). Over Pd-CALB/SPs, this cascade reaction proceeded smoothly and the time for completing the cascade reaction was found to be shorter than the total times of two individual reactions (Supplementary Fig. 31). *R*−1-phenylethyl acetate in 30% yield with 99% ee (enantiomeric access) and 1-phenylethanol in 45% yield with 89% ee, together with 24% ethylbenzene (by-product) were obtained within 6 h. Quite impressively, even after ten reaction cycles the yields and ee values of two products showed no appreciable decrease (for the 10th recycle, 99% ee *R*−1-phenylethyl acetate in 33% yield, and 89% ee 1-phenylethanol in 52% yield). Under the same conditions as applied to Pd-CALB/SPs (Supplementary Table 3), Pd-CALB/MSNs gave 99% ee *R*−1-phenylethyl acetate in 28% yield along with 69% ee 1-phenylethanol in 45% yield (Fig. 5c), which are comparable to the results obtained over Pd-CALB/SPs (the reaction kinetic profiles are included in Fig. 5d and Supplementary Fig. 32). The yields, however, progressively decreased during the consecutive reactions, dropping to 13% for *R*−1-phenylethyl acetate and 42% for 1-phenylethanol in the 4th cycle. The same trend was observed with the assembly of Pd-CALB/MSN to SPs (i.e., Pd-CALB@SPs), where the yields of two products dramatically reduced after four reaction cycles. The catalyst deactivation may be due to the lack of spatial isolation of incompatible Pd NPs and enzymes^49^, since the possibility of the growth of Pd NPs and change in valence state of Pd is excluded (Supplementary Fig. 33 and Supplementary Table 2). Using the physical mixture of Pd/MSNs and CALB/MSNs, the yields and ee values had no significant loss during multiple cycles, confirming the necessity for the spatial separation of Pd NPs and enzymes from each. However, this physically mixed catalyst is much less selective since 16% yield of *R*−1-phenylethyl acetate along with 53% yield of 1-phenylethanol were gotten. This is reasonable because there is a relatively long distance for the intermediate to transfer from Pd NPs to the location of enzymes, where the second step occurs. These results emphasize the advantages of our designed SP-based catalysts, namely a combination of Pd-NPs and enzyme, with each being placed in their own separate locations, while at same time remaining in close proximity.

To obtain the further information on the spatial distance effects, we prepared two other SP-based catalysts by reducing or increasing the MSN sizes (to 60 or 180 nm, Supplementary Fig. 20) while keeping other physical parameters at the same level (Supplementary Fig. 34). These differently sized MSNs lead to different average distances between Pd NPs and CALB. As displayed in Fig. 5e, f, when the MSN size decreased from 100 to 60 nm, the conversion (i.e., the total yields of *R*−1-phenylethyl acetate + 1-phenylethanol + ethylbenzene) dramatically increased, while the total yields of *R*−1-phenylethyl acetate and 1-phenylethanol remarkably decreased (Supplementary Fig. 35). This can be interpreted that reducing the MSN sizes is favorable for the intermediate transfer, thereby leading to timely occurrence of the second step, but the generated final product 1-phenylethyl acetate is in turn converted to the by-product ethylbenzene over Pd NPs (Supplementary Fig. 36). An increase in ee value of 1-phenylethanol was seen, also confirming that reducing the MSN sizes is beneficial to the second step. In contrast, increasing the MSN sizes from 100 to 180 nm resulted in a decrease in the conversion, the total yields as well as the ee value of 1-phenylethanol. This is because the long distance between Pd NPs and CALB is unfavorable to timely transfer of the intermediate. In parallel, we conducted another experiment where the CALB dosage was tuned but the Pd dosage was kept constant (Supplementary Fig. 37, by changing the amount of CALB/MSN). It was found that the conversion and the total yield of *R*−1-phenylethyl acetate and 1-phenylethanol both gradually increased with increasing the CALB dosage despite the fact that CALB does not contributed directly to catalyzing the first step (within the first 2 h). When the reaction proceeded for 4 h, the conversion also increases as the CALB dosage increased but the total yield began to decline due to yielding the by-product. Taken together, in this relatively complex reaction network, two individual steps are seen to interfere with each other through spatiotemporal effects arising from SPs, even though Pd and enzyme are exclusively active toward hydrogenation or kinetic resolution.

To test the potential for practical applications of the SP-based catalysts, we packed Pd-CALB/SPs in a fixed-bed reactor for continuous flow catalysis. A solution of acetophenone was continuously pumped into the inlet of this reactor and the product-containing stream was collected from its outlet. As Fig. 5g displays, the yields of *R*−1-phenylethyl acetate and 1-phenylethanol were 18–21% and 46–53% in spite of slight fluctuations. A *R*−1-phenylethyl acetate with 99% ee and 1-phenylethanol with 70–86% ee were maintained. After running for 200 h, the yields and ee values did not significantly decline albeit at the expense of the flow rate. We found that the conversion over Pd-CALB/SPs is much higher than that obtained in the two-stage process with dual bed arranged Pd/SPs and CALB/SPs (Supplementary Fig. 38). This can be ascribed to the shorter distance between different catalytic species, which is favorable to timely transfer of the intermediate. The morphology, size and porous structures of the SPs were essentially unchanged (Fig. 4n and Supplementary Fig. 39). 86% of the initial Pd and 83% of the initial protein enzyme were retained (Supplementary Table 1). No significant Pd leaching could be detected in the reactions, and severe Pd aggregations were not observed (Supplementary Fig. 40). For the other substrates such as *p*-methylacetophenone, the total yields of 1-(4-methylphenyl)ethyl acetate and 1-(4-methylphenyl)ethanol were maintained at the levels of 75% over a period of 300 h; the corresponding esters with 99% ee and alcohols with 85–97% ee were lasted during this course (Fig. 5h). Furthermore, to highlight the importance of our SP-based method in the synthesis of chiral esters, we prepared another SPs-based catalyst (denoted as H-beta-CALB/SPs) by combining H-beta zeolite and lipase CALB-immobilized MSNs together (Supplementary Fig. 41), which exhibited more than 97% ee of chiral ester during 800 h of continuous flow reaction and enhanced catalytic efficiency in comparison to its counterpart catalysts (Supplementary Figs. 42 and 43).

### Cascade of amine racemization and kinetic resolution over Pd-CALB/SPs

**Fig. 6:**
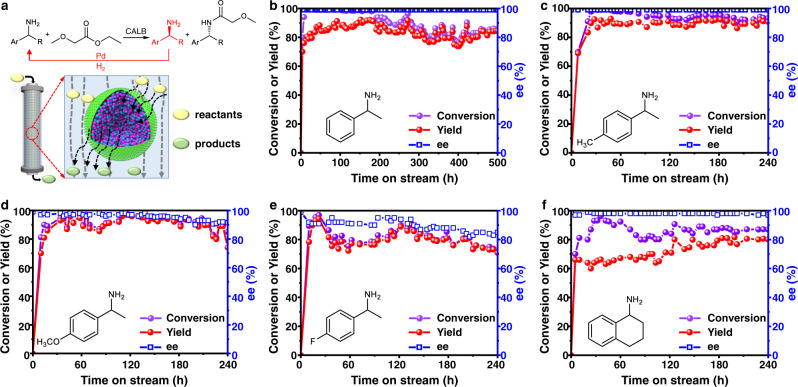
Results of the cascade of amine racemization and kinetic resolution over Pd-CALB/SPs in continuous flow systems. Reaction conditions: 0.85 g Pd-CALB SPs packed in a fixed-bed reactor, 0.1 M racemic amine in toluene, 0.2 M ethyl methoxyacetate in toluene, 70 °C, 1 MPa (95% N_2_/5% H_2_, 10 mL min^−1^). **a** Reaction network of the cascade reaction. **b** 1-phenylethylamine, flow rate from initial 1.2 mL h^−1^ to 0.6 mL h^−1^ at the end. **c** 1-(4-methylphenyl)ethylamine, flow rate from initial 1.2 mL h^−1^ to 0.6 mL h^−1^ at the end. **d** 1-(4-methoxyphenyl)ethylamine, flow rate from initial 1.2 mL h^−1^ to 0.6 mL h^−1^ at the end. **e** 1-(4-fluorophenyl)ethylamine, flow rate from initial 1.2 mL h^−1^ to 0.6 mL h^−1^ at the end. **f** 1,2,3,4-tetrahydro-1-naphthylamine, flow rate from initial 1.2 mL h^−1^ to 0.5 mL h^−1^ at the end.

To further check the potential of the SP-based catalyst in practical applications, we evaluated Pd-CALB/SPs with another cascade reaction, which comprises two steps: enzymatic kinetic resolution of amines and in situ Pd-catalyzed racemization of amine (Fig. 6a)^54–57^. Although tremendous effort has been made^56,57^, the design of catalysts for this cascade reaction still faces critical challenges, including unsatisfactory recyclability, and infeasibility of use in fixed-bed reactors.

Interestingly, Pd-CALB/SPs worked very well in fixed-bed reactors. Over a period of 500 h (1 MPa 95% N_2_/5% H_2_, at 70 °C), the conversion of 1-phenethylamine was always more than 85%, and 75–92% yield of the product with 99% ee was obtained (Fig. 6b). A small amount of ethylbenzene as side-product was observed (<15%). Its turnover number (TON, in terms of Pd) reached 827 in terms of Pd, far exceeding TONs of the best currently existing catalysts^49,54–57^. After such a long period of reaction, Pd-CALB/SPs seems intact in terms of spherical shape, without an apparent decrease in the mechanical strength (Supplementary Fig. 44). 85% Pd and 72% protein were retained in Pd-CALB/SPs despite such a long period of operation (Supplementary Table 4). The experiment with the eluent from the reactor further confirms a negligible level of enzyme leaching since the eluent exhibited no enzymatic activity (Supplementary Fig. 45). The excellent activity and exceptional stability of Pd-CALB/SPs should be attributed to its structural characteristic, namely the spatial separation but retention of close proximity of the two different catalytic sites. Moreover, for 1-(4-methylphenyl)ethylamine, ee value of 99% for amide was also maintained over a course of 240 h and the yield of the corresponding amide were always >91% (Fig. 6c). For less reactive substrates such as 1-(4-methoxyphenyl)ethylamine, and 1,2,3,4-tetrahydro-1-naphthylamine (Fig. 6d, f), ee values of 92–99% of amides in >66% yield were achieved over 240 h. Moreover, for 1-(4-fluorophenyl)ethanamine, an ee value of 82% for amide was also maintained over a course of 240 h and the yield of the corresponding amide was always >70% (Fig. 6e). Collectively, these results highlight the potential utilization of our cascade catalyst in practical applications.

## Discussion

We demonstrated a new method to prepare robust mm-sized spherical SPs through a liquid marble route with the aid of strength additives or interparticle interactions, and further illustrate the use of this method to fabricate SP-based biomimetic cascade catalysts. The key to such a success is the assembly of NPs within the confined spaces of liquid marbles, which makes it possible to prepare large microspheres without the need for special substrate surfaces or specific liquid compositions that are required by the existing methods. The yielded SPs exhibit a tunable porous structure, and a mechanical strength that can be even comparable to that for calcined counterparts. The exceptional mechanical strength arises from the strong hydrogen-bonding interactions between amine and silanols on the silica nanoparticle surfaces, which assist to hold the NPs together. This bottom-up protocol was shown to be feasible to fabricate cascade catalysts using well-defined catalytically active NPs, as exemplified here by a Pd-enzyme cascade catalyst. Without the need for the high temperature calcination, our protocol should be particularly effective for the assembly of catalysts that are unstable during high temperature treatment. With their mm-levelled size and high robustness, the developed SP-based catalysts could be directly packed in the industrially preferred fixed-bed reactors for continuous-flow chiral catalysis, and exhibits good activity and long-term stability in two distinct cascade reactions (200 h or 500 h). The excellent performance is attributed to the unique feature of the SP-based cascade catalyst that reconciles both spatial separation of two different types of catalytically active NPs and their proximity. Importantly, the parameters of the spatial separation and proximity that significantly impact the cascade reaction kinetics, can be rationally tuned by adjusting the sizes of catalytically active NPs and their ratio. We believe that this study opens a new avenue in the design of industrially relevant cascade catalysts in a controllably biomimetic fashion since the method reported here is straightforward but enables a bottom-up assembly of well-defined catalytically active particles into robust large spherical particles under mild conditions.

## Methods

### Preparation of supraparticles (SPs)

Typically, MSNs were added to an aqueous TEPA solution, leading a suspension consisting of 4 wt% TEPA, 10 wt% MSNs and 86 wt% water. After sufficiently mixing, this suspension was transferred into a syringe with an orifice diameter of 0.5 mm and then continuously dropped onto a bed made of hydrophobic spongy porous silica followed by rolling on the bed, generating liquid marbles. The resultant liquid marbles were then transferred onto a piece of paper. The water inside the resultant marbles was slowly evaporated under ambient conditions (25 °C), generating the desired SPs.

### Preparation of SP-based cascade catalyst (Pd-CALB/SPs)

Typically, 0.5 g Pd/MSNs and 0.5 g CALB/MSNs were added to 9 g water, forming a suspension via a sufficient mixing. This suspension was transferred into a syringe with an orifice diameter of 0.5 mm and then continuously dropped onto a bed made of hydrophobic spongy porous silica followed by rolling on the bed, forming liquid marbles. The resultant liquid marbles were then transferred onto a piece of paper for slow evaporation of water under ambient conditions (25 °C), eventually producing a SP-based catalyst, i.e., Pd-CALB/SPs.

## Supplementary information

Supplementary Information

## Data Availability

The data for Figs. 2–6 generated in this study are provided in the Supplementary Information/Source Data file. Source data are provided with this paper.

## Source data

Source Data
