# Supplementary material for: A supraparticle-based biomimetic cascade catalyst for continuous flow reaction
Source: Nat Commun. 2022 Oct 8;13:5935. doi: 10.1038/s41467-022-33756-1 (PMC9547976; doi:10.1038/s41467-022-33756-1)
Supplement: Supplementary file 1 — Supplementary Information [file 41467_2022_33756_MOESM1_ESM.pdf]

Supplementary Information for

A supraparticle-based biomimetic cascade catalyst for continuous flow reaction

Xiaomiao Guo[1,†], Nan Xue[1,†], Ming Zhang[1], Rammile Ettelaie[2] and Hengquan Yang[1,3]*

[1]*School of Chemistry and Chemical Engineering, Shanxi University, Taiyuan 030006, China*

[2]*Food Colloids Group, School of Food Science and Nutrition, University of Leeds, Leeds LS2 9JT, U.K.*

[3]*Key Laboratory of Chemical Biology and Molecular Engineering of Ministry of Education, Shanxi University, Taiyuan 030006, China*

[†]*These authors contributed equally: Xiaomiao Guo, Nan Xue*

*To whom correspondence should be addressed: hqyang@sxu.edu.cn

Contents

Supplementary Figure 1. TG curves of bare drops and liquid marbles
Supplementary Figure 2. Characterization of spongy porous silica and octyl-modified spongy porous silica
Supplementary Figure 3. Characterization of mesoporous silica nanospheres (MSNs)
Supplementary Figure 4. Appearance of bare drops before and after evaporation
Supplementary Figure 5. Appearance of SPs prepared at higher evaporation temperatures for observing the evaporation rate effects
Supplementary Figure 6. SEM image showing the shell of SPs
Supplementary Figure 7. Time-dependent fluorescence intensity for the transport of Rhodamine B into a supraparticle
Supplementary Figure 8. Appearance of SPs with different particle sizes
Supplementary Figure 9. Interior structures of SPs after calcination
Supplementary Figure 10. Appearance of SPs after the treatment with various solvents in a fixed-bed reactor
Supplementary Figure 11. SEM images of SPs after fluidization treatment
Supplementary Figure 12. Appearance and SEM images of SPs after the test in a fluidized-bed reactor or fixed-bed reactor
Supplementary Figure 13. Appearance of SPs prepared with different amounts of TEPA
Supplementary Figure 14. Viscosity of aqueous TEPA solution as a function of TEPA concentration
Supplementary Figure 15. Appearance of SPs prepared with different strength additives
Supplementary Figure 16. Appearance of SPs prepared with different strength additives
Supplementary Figure 17. Appearance of the liquid marble in the absence of MSNs
Supplementary Figure 18. Characterization of SPs prepared with different amounts of MSNs
Supplementary Figure 19. SEM images of MSNs with different particle sizes
Supplementary Figure 20. SEM images showing the interior structure of SPs prepared with differently sized MSNs
Supplementary Figure 21. N_2 sorption measurement results of SPs prepared with different amounts of TEPA
Supplementary Figure 22. N_2 sorption measurement results of SPs prepared with different amounts of MSNs
Supplementary Figure 23. N_2 sorption measurement results of SPs prepared with differently sized MSNs
Supplementary Figure 24. Characterization of SPs prepared with different building blocks
Supplementary Figure 25. Mechanical strength for SPs prepared with various organo-modified MSNs or CALB-immobilized MSNs
Supplementary Figure 26. Characterization of MSNs, amine-modified MSNs and octyl-modified MSNs
Supplementary Figure 27. Characterization of MSNs, amine-modified MSNs and octyl-modified MSNs
Supplementary Figure 28. N_2 sorption measurement results of amine-modified MSNs and octyl-modified MSNs
Supplementary Figure 29. N_2 sorption measurement results of Pd/MSNs and CALB/MSNs

Supplementary Figure 30. Fluorescence confocal microscopy images of SPs prepared with MSNs
Supplementary Figure 31. Kinetic profiles of individual step and cascade reactions
Supplementary Figure 32. Kinetic profiles of cascade of ketone hydrogenation and alcohols kinetic resolution over different catalysts in batch reactions
Supplementary Figure 33. Characterization of Pd-CALB@SPs
Supplementary Figure 34. N_2 sorption measurement results of Pd-CALB/SPs prepared with differently sized MSNs
Supplementary Figure 35. Reaction results of sequential hydrogenation-kinetic resolution of acetophenone over SPs prepared with differently sized MSNs
Supplementary Figure 36. Kinetic profiles of 1-phenylethanol hydrogenation and 1-phenylethyl acetate hydrogenation over Pd-CALB/SPs
Supplementary Figure 37. Results of sequential hydrogenation-kinetic resolution of acetophenone over SPs prepared with different mass ratios of Pd/MSNs to CALB/MSNs
Supplementary Figure 38. Results of the cascade of hydrogenation and kinetic resolution over Pd/SPs and CALB/SPs in two-stage reactions
Supplementary Figure 39. N_2 sorption measurements results of Pd-CALB/SPs after 200 h of continuous flow reaction
Supplementary Figure 40. Characterization of Pd-CALB/SPs after 200 h of continuous flow reaction
Supplementary Figure 41. Characterization of H-beta-CALB/SPs
Supplementary Figure 42. Results of the cascade reaction for the synthesis of chiral 1-phenylethanol acetate over different catalysts
Supplementary Figure 43. Results of the cascade reaction for the synthesis of chiral 1-phenylethanol acetate over H-beta-CALB/SPs in a continuous flow system
Supplementary Figure 44. Characterization of Pd-CALB/SPs after 500 h of continuous flow reaction
Supplementary Figure 45. Results of testing CALB activity in the eluent from sequential amine racemization-kinetic resolution
Supplementary Table 1. Results of ICP-MS analysis of Pd-CALB/SPs before and after 200 h of continuous flow reaction
Supplementary Table 2. Results of ICP-MS analysis of Pd-CALB@SPs before and after four reaction recycles
Supplementary Table 3. Loadings of Pd and CALB in various catalysts
Supplementary Table 4. Results of ICP-MS analysis of Pd-CALB/SPs before and after 500 h of continuous flow reaction

Supplementary Methods

Mass Spectrometry Spectra

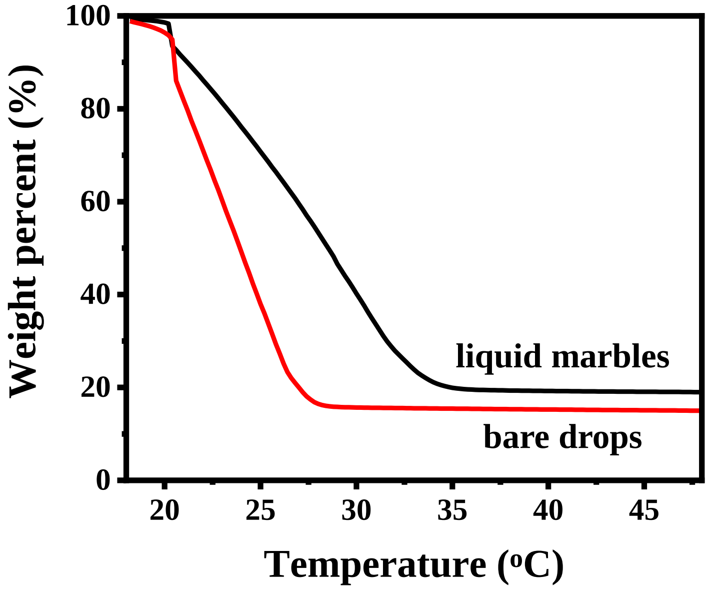

Supplementary Figure 1. TG curves of bare drops and liquid marbles. Bare drops and liquid marbles were prepared with 4 wt% TEPA and 10 wt% MSNs (with respect to the total mass of water, TEPA and MSNs), otherwise was mentioned in. The TG analysis was performed under air with a heating rate of 0.2 °C min^{-1}.

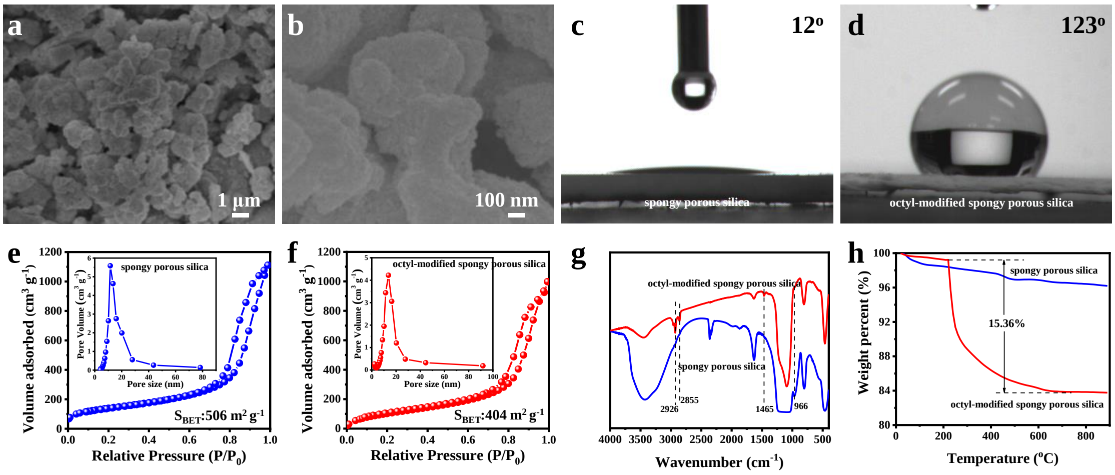

Supplementary Figure 2. Characterization of spongy porous silica and octyl-modified spongy porous silica. a, SEM image of spongy porous silica. **b**, Magnified SEM image of spongy porous silica. **c**, Appearance of a water droplet in air on a disk of compressed spongy porous silica and the corresponding contact angle. **d**, Appearance of a water droplet in air on a disk of compressed octyl-modified spongy porous silica and the corresponding contact angle. **e**, N_2 adsorption-desorption isotherm and BJH pore size distribution of spongy porous silica (inset). **f**, N_2 adsorption-desorption isotherm and BJH pore size distribution of octyl-modified spongy porous silica (inset). **g**, FT-IR spectra of spongy porous silica and octyl-modified spongy porous silica. **h**, TG curves of spongy porous silica and octyl-modified spongy porous silica.

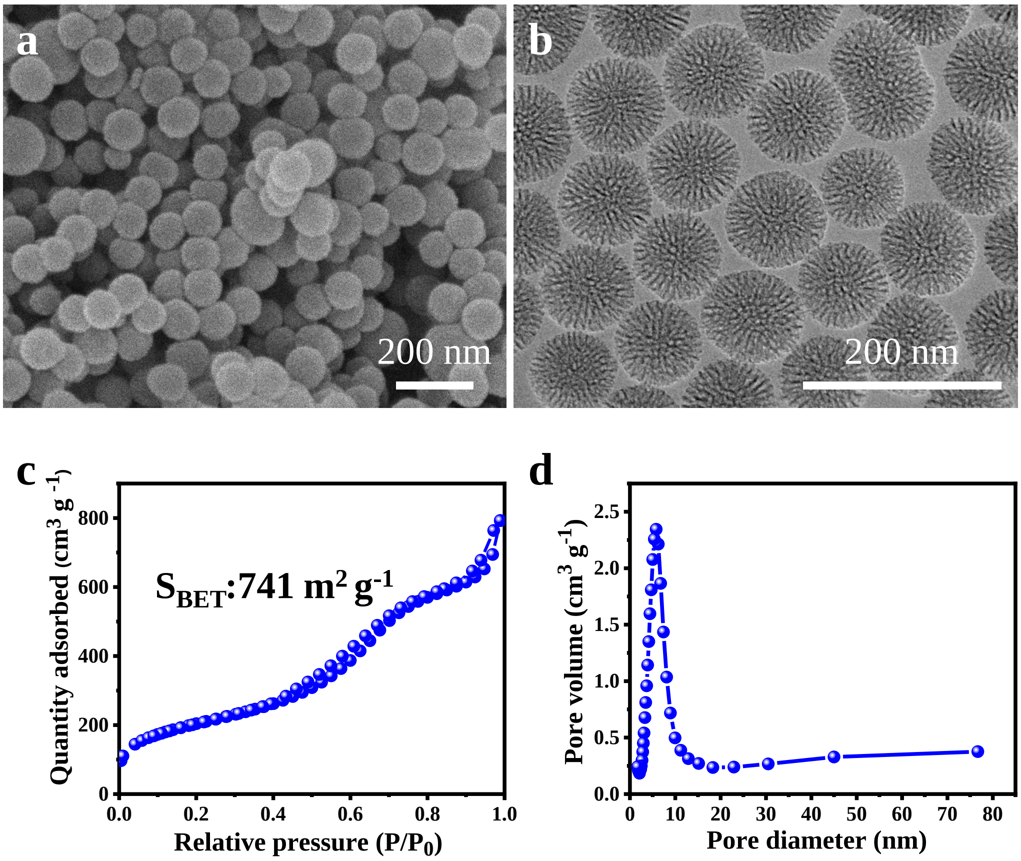

Supplementary Figure 3. Characterization of mesoporous silica nanospheres (MSNs). a, SEM image. **b**, TEM image. **c**, N_2 adsorption-desorption isotherm. **d**, BJH pore size distribution.

Notes: The average diameter of MSNs is *ca*. 100 nm, measured by TEM. The BET specific surface area was measured to be 741 m^2 g^{-1} and the average pore size is *ca*. 5.7 nm, calculated from the BJH method.

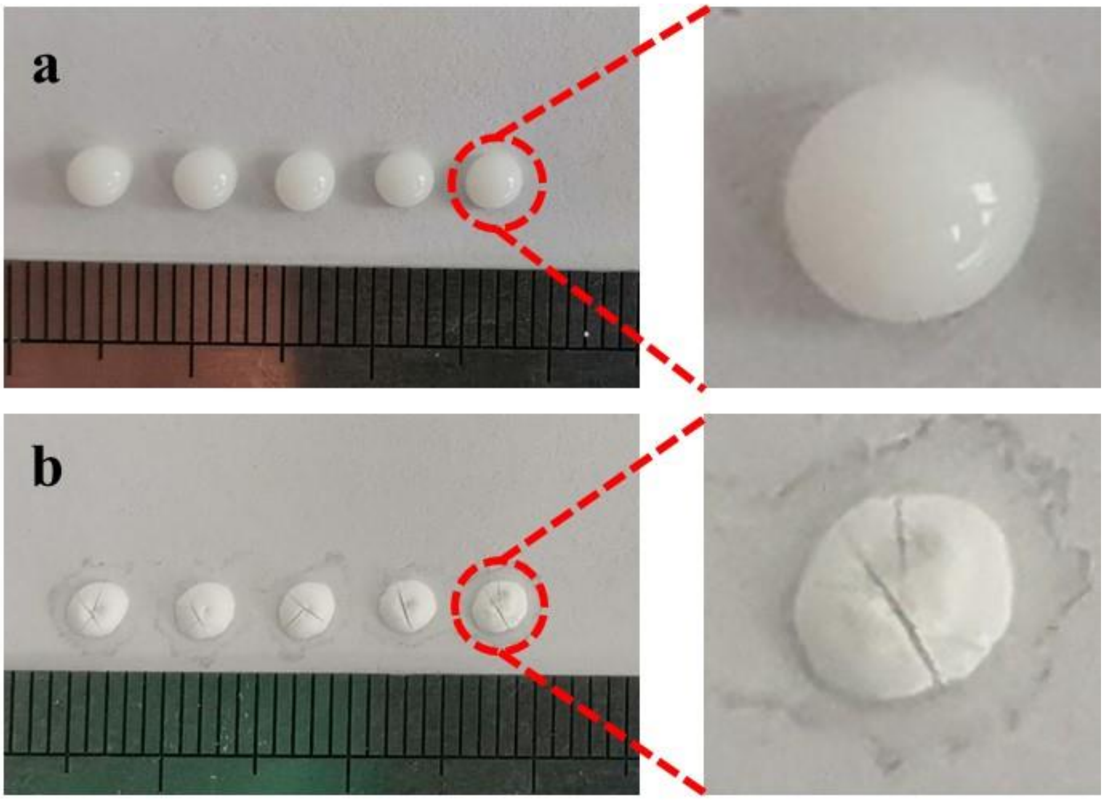

Supplementary Figure 4. Appearance of bare drops before and after evaporation (without adsorption of octyl-modified spongy mesoporous silica, containing 4 wt% TEPA and 10 wt% MSNs with respect to the total mass of water, TEPA and MSNs). **a**, Appearance of the bare drops before evaporation (under ambient conditions). **b**, Appearance of the bare drops after evaporation.

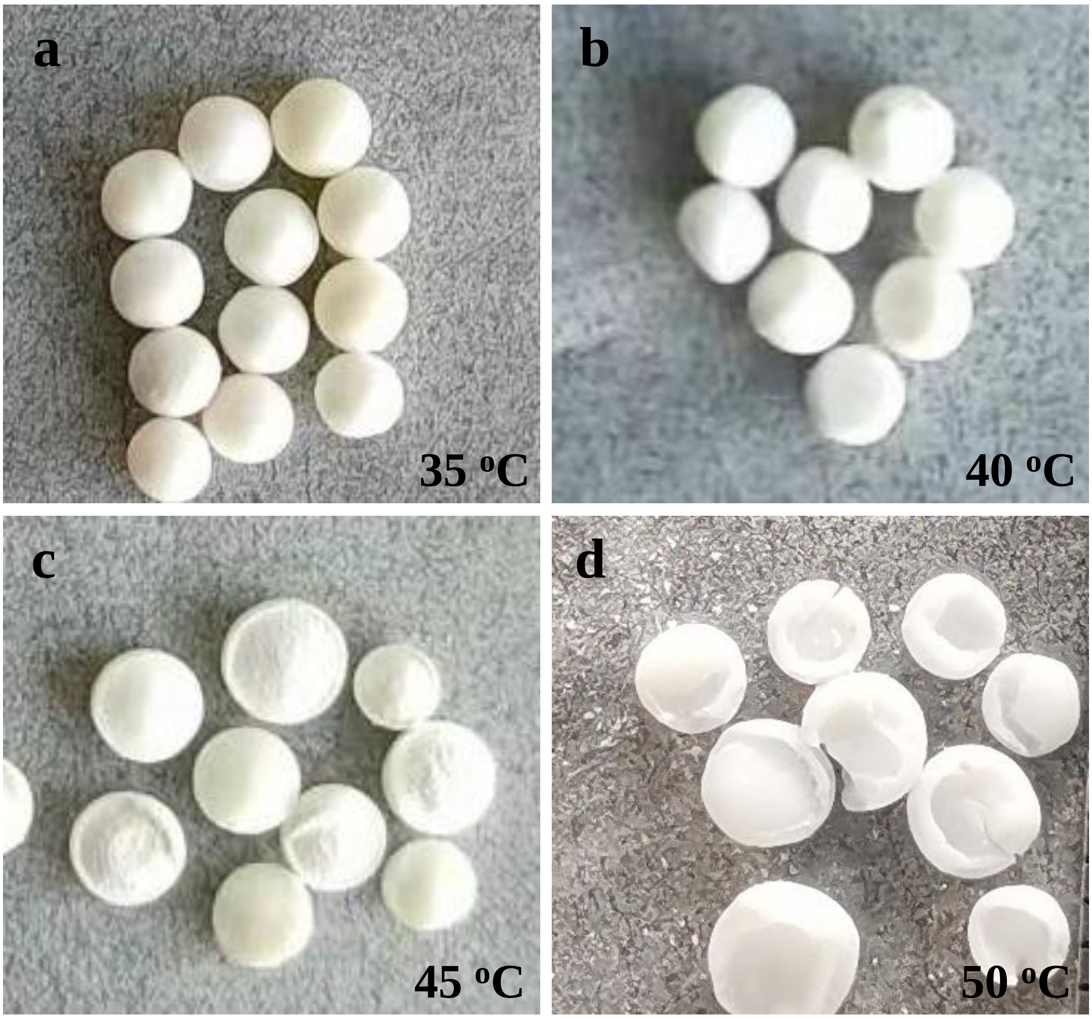

Supplementary Figure 5. Appearance of SPs prepared at higher evaporation temperatures for observing the evaporation rate effects. **a**, 35 ºC. **b**, 40 ºC. **c**, 45 ºC. **d**, 50 ºC.

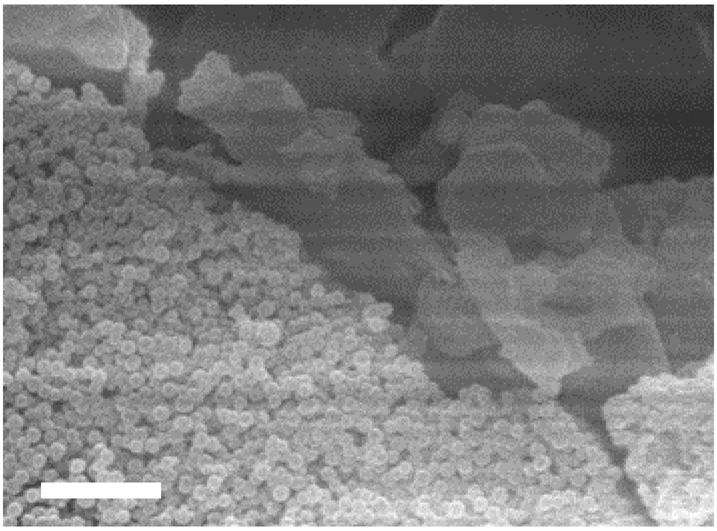

Supplementary Figure 6. SEM image showing the shell of SPs. Scale bars = 1 μm.

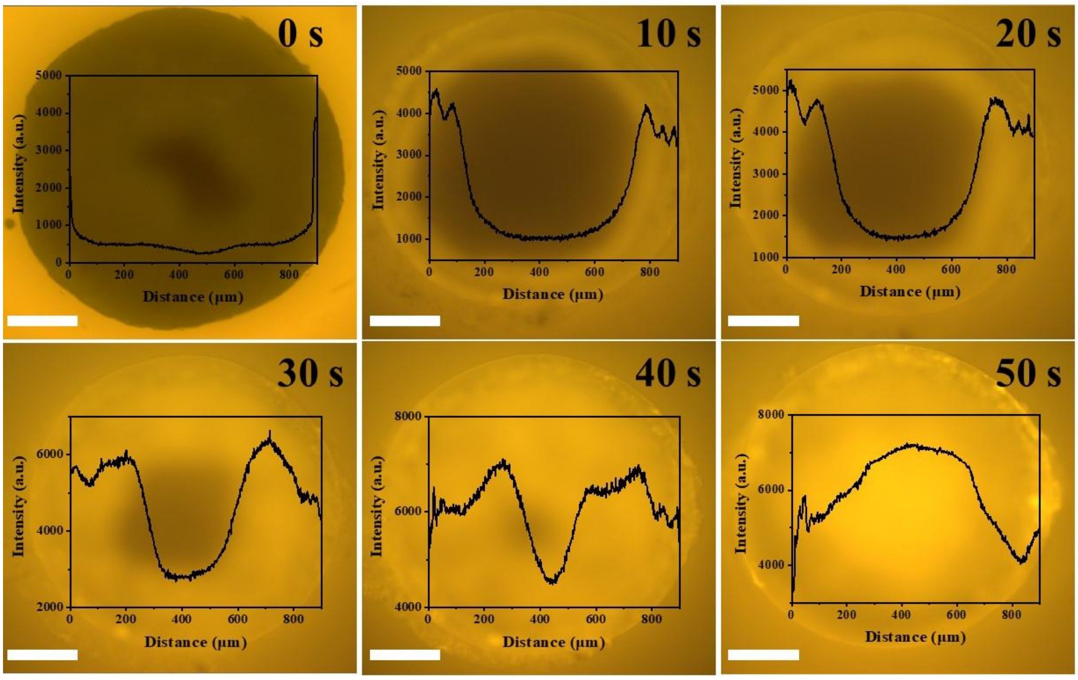

Supplementary Figure 7. Time-dependent fluorescence intensity for the transport of Rhodamine B into a supraparticle. Scale bars = 200 μm.

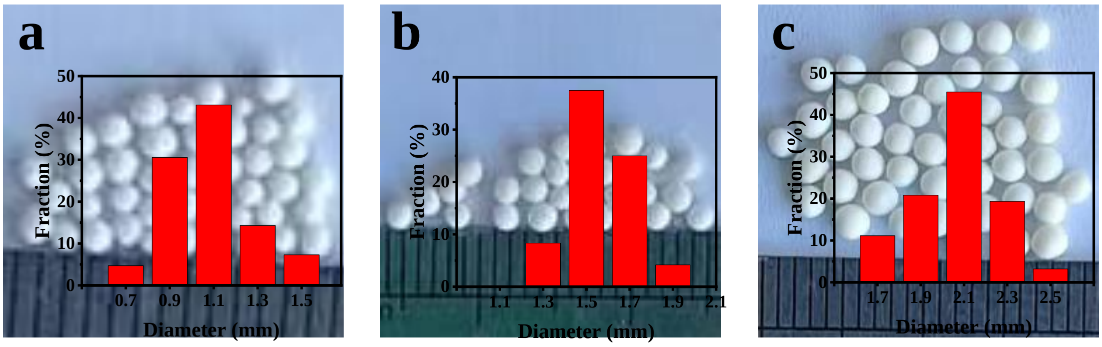

Supplementary Figure 8. Appearance of SPs with different particle sizes. The size of SPs was tuned through variation of feeding rate of the syringe pump in combination with the inner diameter of the syringe. **a**, 1.1 mm (flow rate: 0.5 mL min^{-1}; the inner diameter: 0.3 mm). **b**, 1.5 mm (flow rate: 0.3 mL min^{-1}; inner diameter: 0.5 mm). **c**, 2.1 mm (flow rate: 0.1 mL min^{-1}; inner diameter: 0.7 mm).

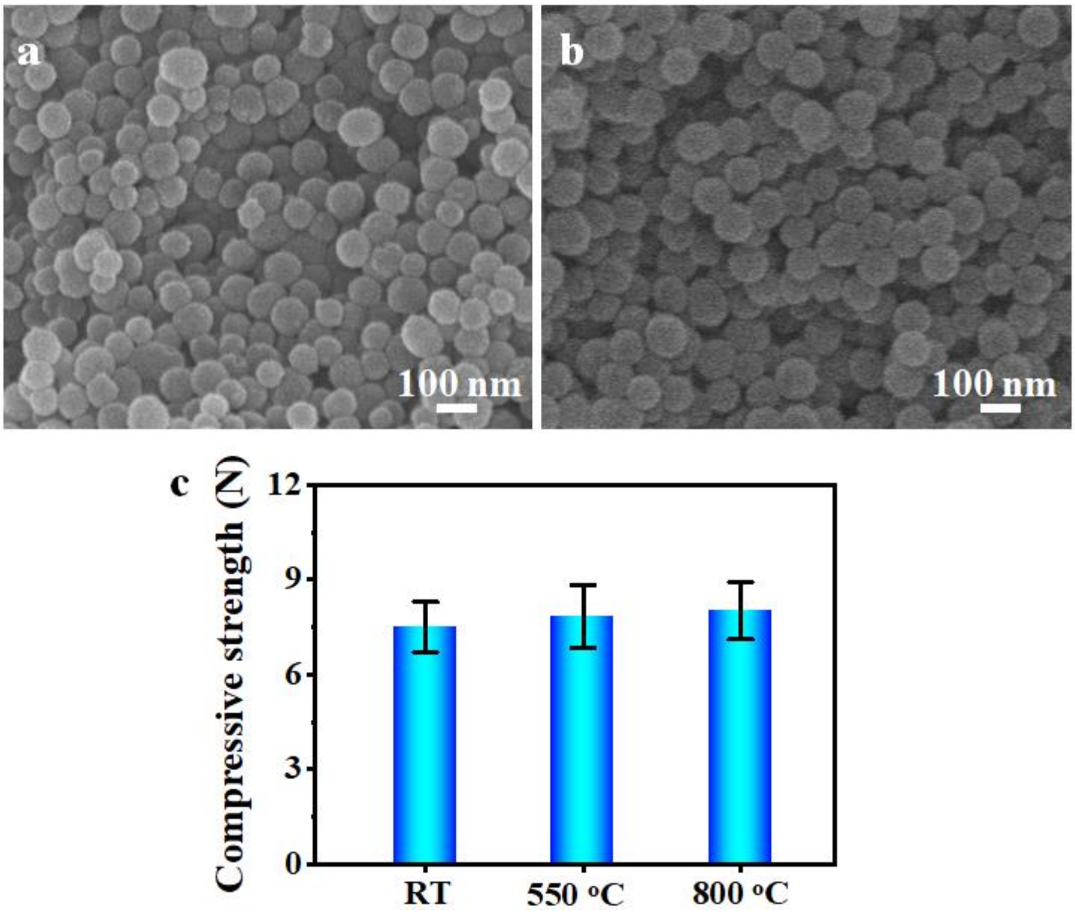

Supplementary Figure 9. Interior structures of SPs after calcination. **a**, SEM image showing the interior structures of SPs after calcination at 550 ^{o}C. **b**, SEM image showing the interior structures of SPs after calcination at 800 ^{o}C. **c**, Mechanical strength for SPs before and after calcination at different temperatures. Error bars indicate the standard error from 20 samples averages.

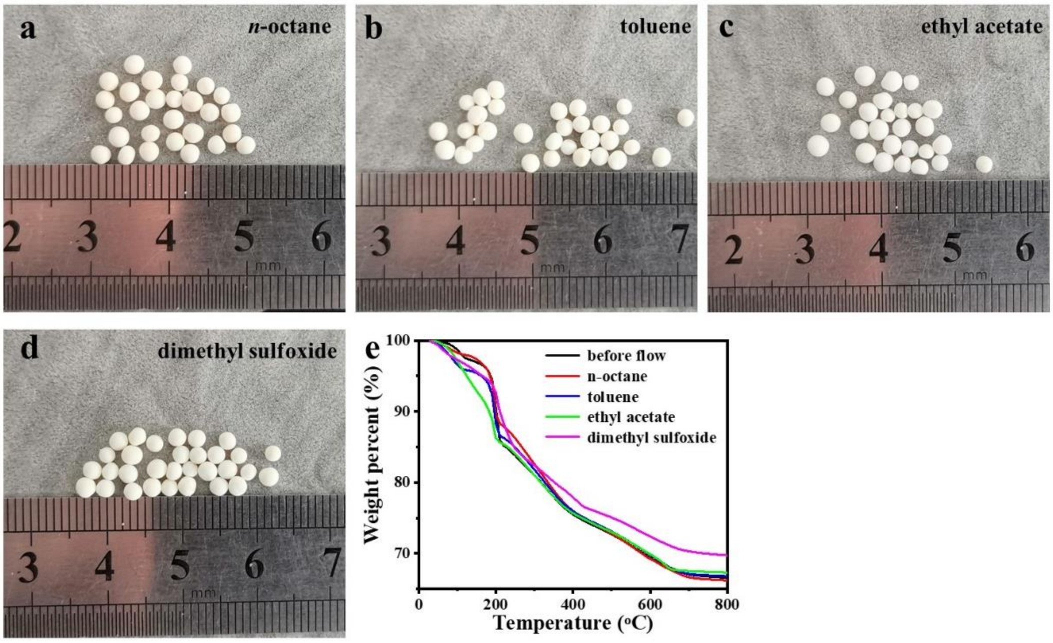

Supplementary Figure 10. Appearance of SPs after the treatment with various solvents in a fixed-bed reactor (48 h, 20 mL min^{-1}). **a**, *n*-octane. **b**, toluene. **c**, ethyl acetate. **d**, dimethyl sulfoxide (DMSO). **e**, TGA results of the SPs before and after the test.

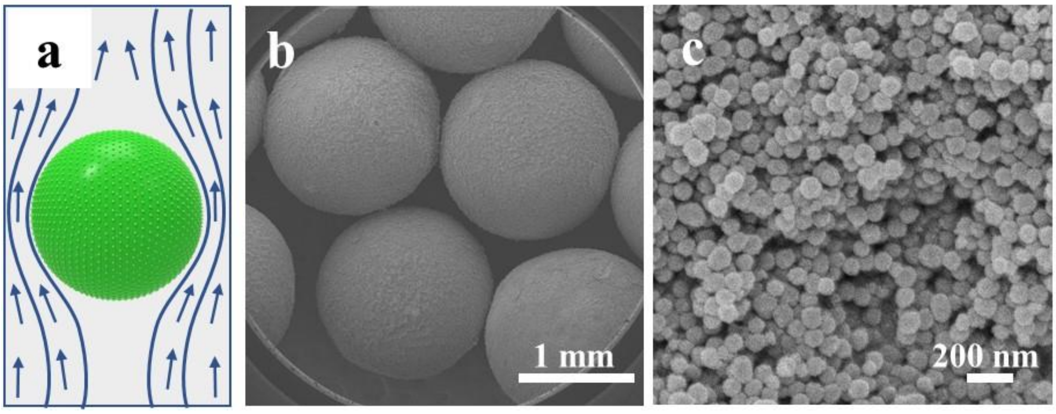

Supplementary Figure 11. SEM images of SPs after fluidization treatment. **a**, Schematic illustration of SPs in a fluidized-bed reactor. **b**, SEM image showing the morphology after the test (Fluidization conditions: N_2 at a superficial velocity ca. 0.3 m s^{-1}). **c**, SEM image showing the interior structure of SPs after the test.

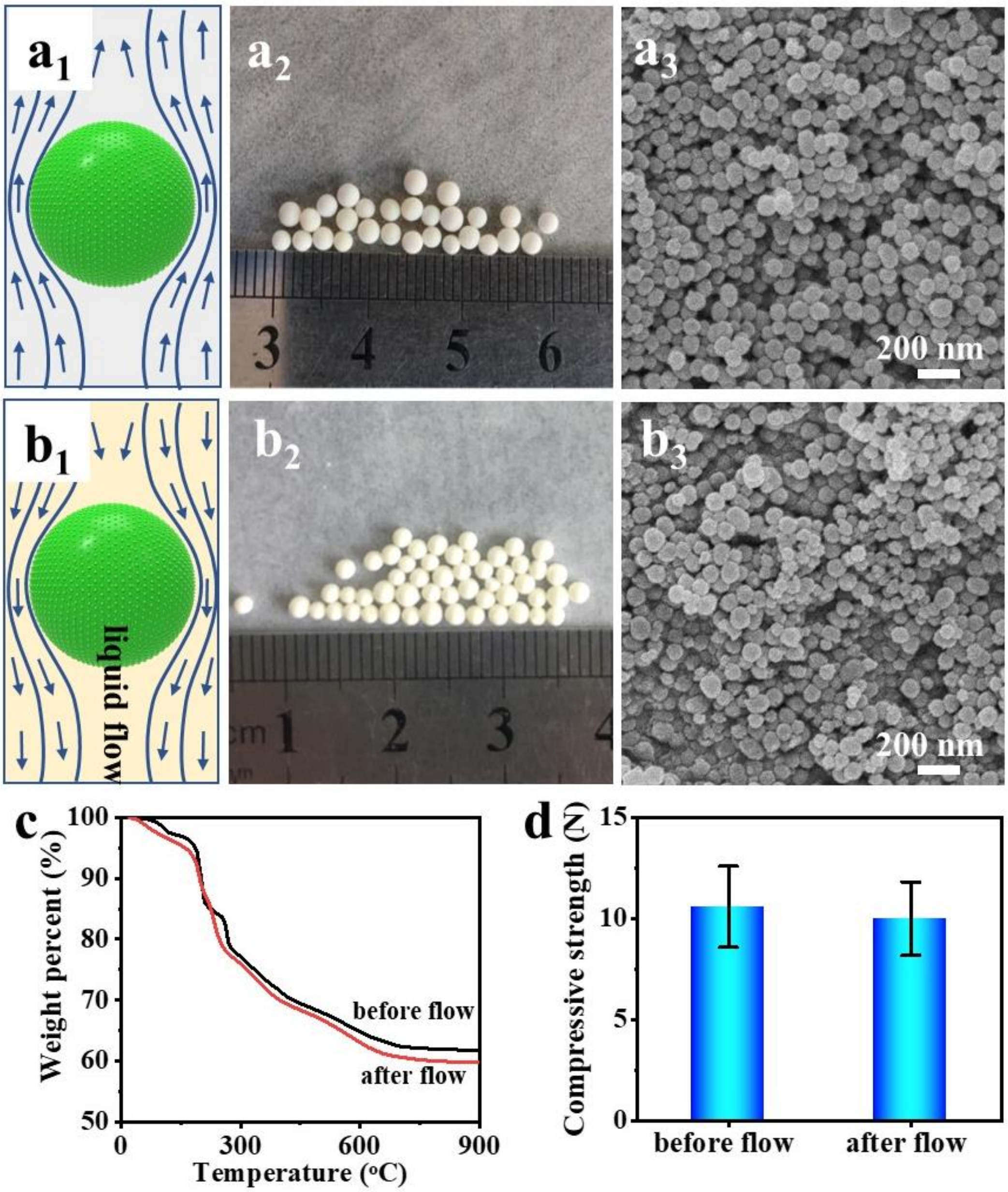

Supplementary Figure 12. Appearance and SEM images of SPs after the test in a fluidized-bed reactor or fixed-bed reactor (here SPs were prepared 20 wt% MSNs and 4 wt% TEPA). **a**1, Schematic illustration of SPs in a fluidized-bed reactor. **a**2 and **a**3, Appearance and SEM images of SPs after the test. Fluidization conditions: N_2 at a superficial velocity ca. 0.3 m s^{-1}. **b**1, Schematic illustration of SPs in a fixed-bed reactor. **b**2 and **b**3, Appearance and SEM images of SPs after the test. Treatment conditions: *n*-octane at 4 MPa, 6 mL h^{-1}. **c**, TG curves of the SPs before and after the test. **d**, Mechanical strength for SPs before and after the treatment. Error bars indicate the standard error from 20 samples averages.

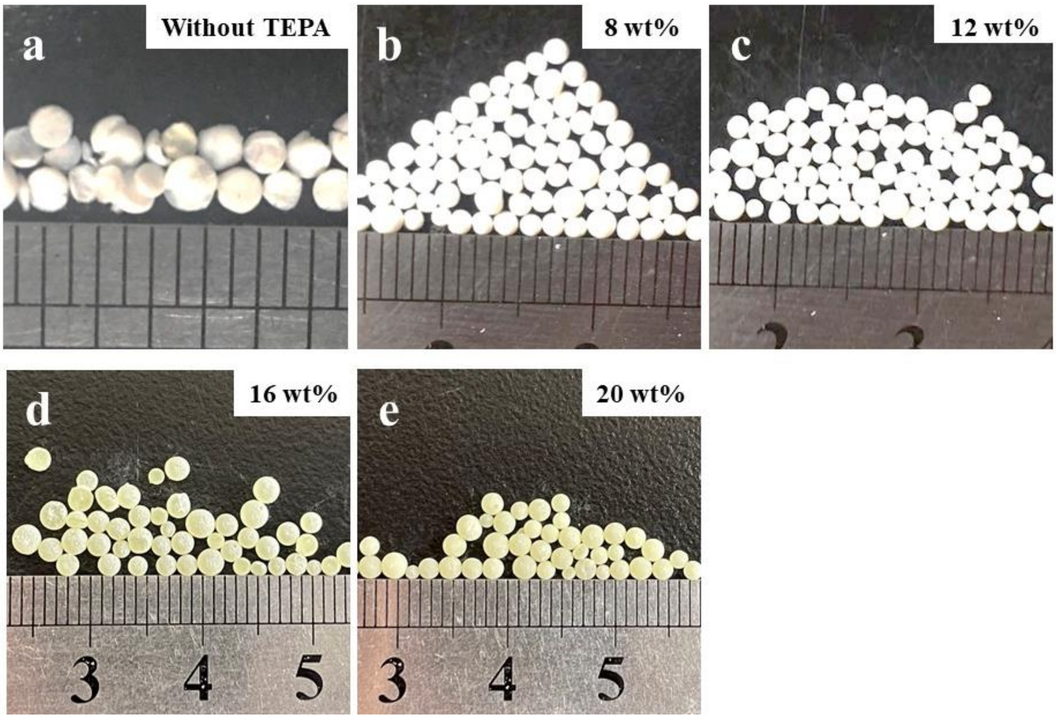

Supplementary Figure 13. Appearance of SPs prepared with different amounts of TEPA (keeping the amount of MSNs constant at 10 wt%). **a**, Without TEPA. **b**, 8 wt%. **c**, 12 wt%. **d**, 16 wt%. **e**, 20 wt%.

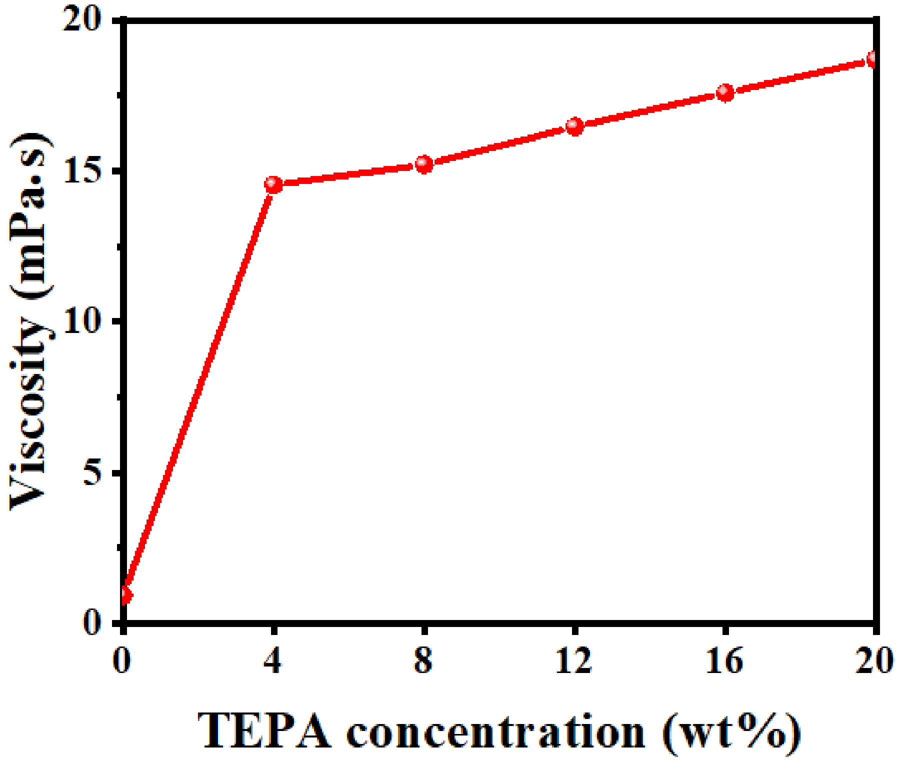

Supplementary Figure 14. Viscosity of aqueous TEPA solution as a function of TEPA concentration. The viscosity of the solutions at different concentrations of TEPA was measured with Rotary Viscometer at 25 °C.

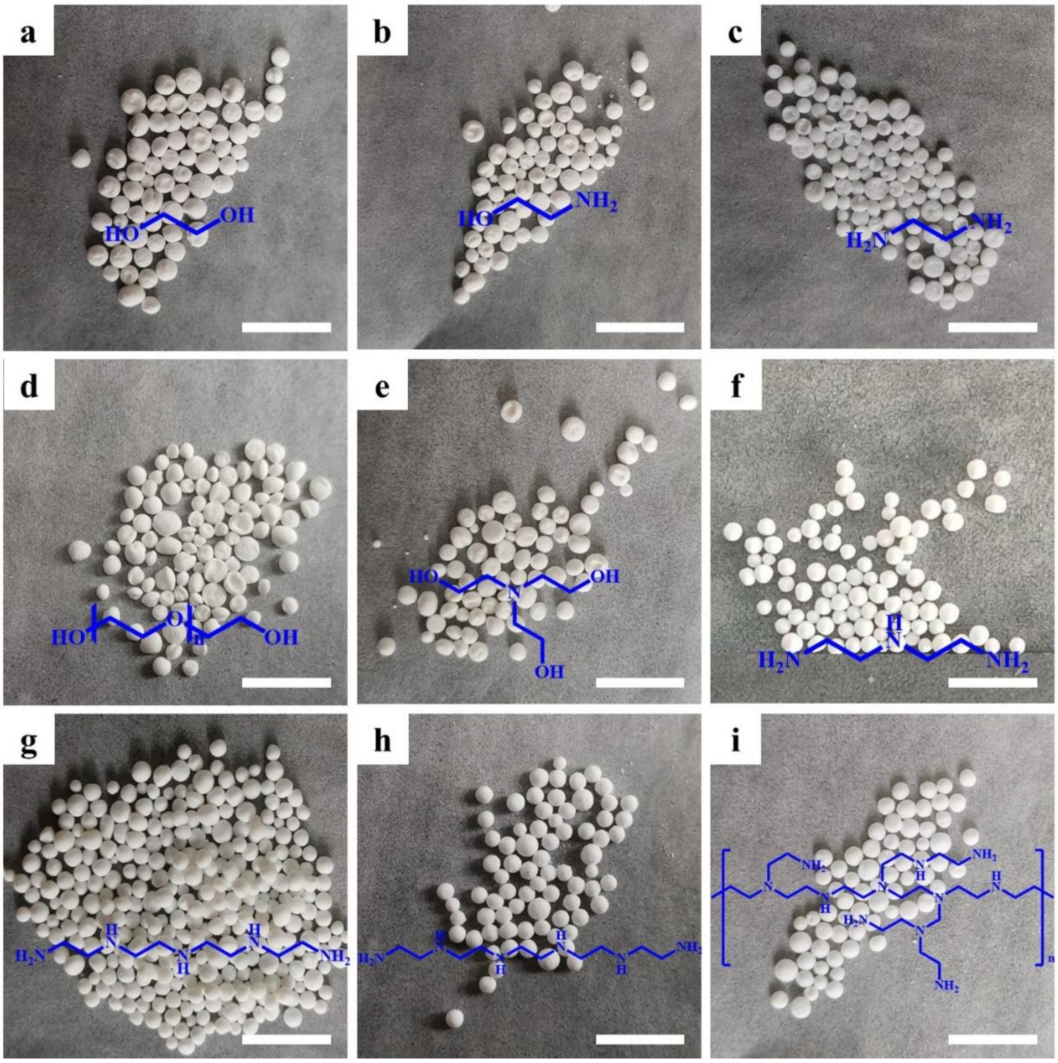

Supplementary Figure 15. Appearance of SPs prepared with different strength additives (keeping the amounts of strength additives and MSNs at 4 wt% and 10 wt%). **a**, ethylene glycol ($HO(CH_2)_2OH$). **b**, ethanolamine ($HO(CH_2)_2NH_2$); **c**, ethylenediamine ($H_2N(CH_2)_2NH_2$). **d**, polyethylene glycol ($HO(CH_2CH_2O)_nH$). **e**, triethanolamine ($(HOCH_2CH_2)_3N$). **f**, diethylenetriamine ($NH_2(CH_2)_2NH(CH_2)_2NH_2$). **g**, tetraethylenepentamine ($H_2N(CH_2CH_2NH)_3CH_2CH_2NH_2$). **h**, pentaethylenehexamine ($H_2N(CH_2CH_2NH)_4CH_2CH_2NH_2$). **i**, polyethyleneimine ($(CH_2CH_2NH)_n$). Scale bars = 1 cm.

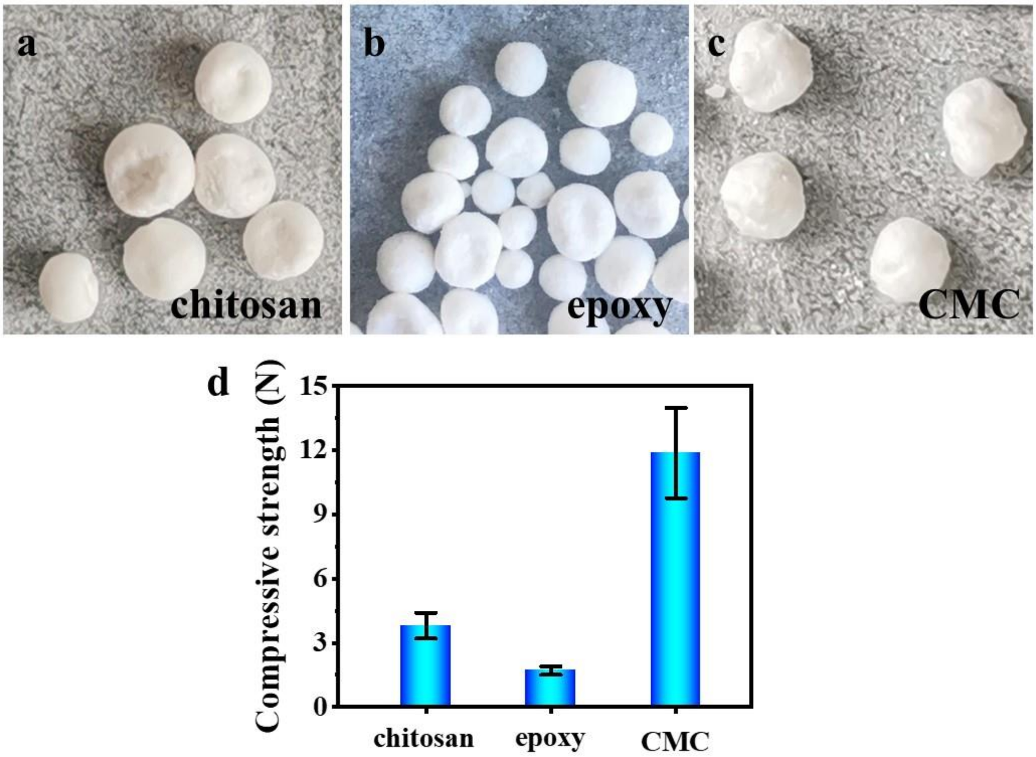

Supplementary Figure 16. Appearance of SPs prepared with different strength additives (keeping the amounts of strength additives and MSNs at 4 wt% and 10 wt%). **a**, Chitosan; **b**, Epoxy; **c**, Carboxymethylcellulose (CMC). **d**, Mechanical strength of SPs. Error bars indicate the standard error from 20 samples averages.

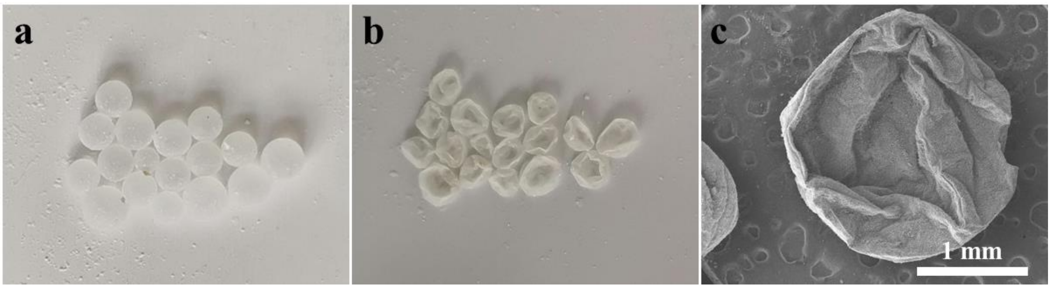

Supplementary Figure 17. Appearance of the liquid marble in the absence of MSNs. a, Before evaporation. **b**, After evaporation. **c**, SEM image.

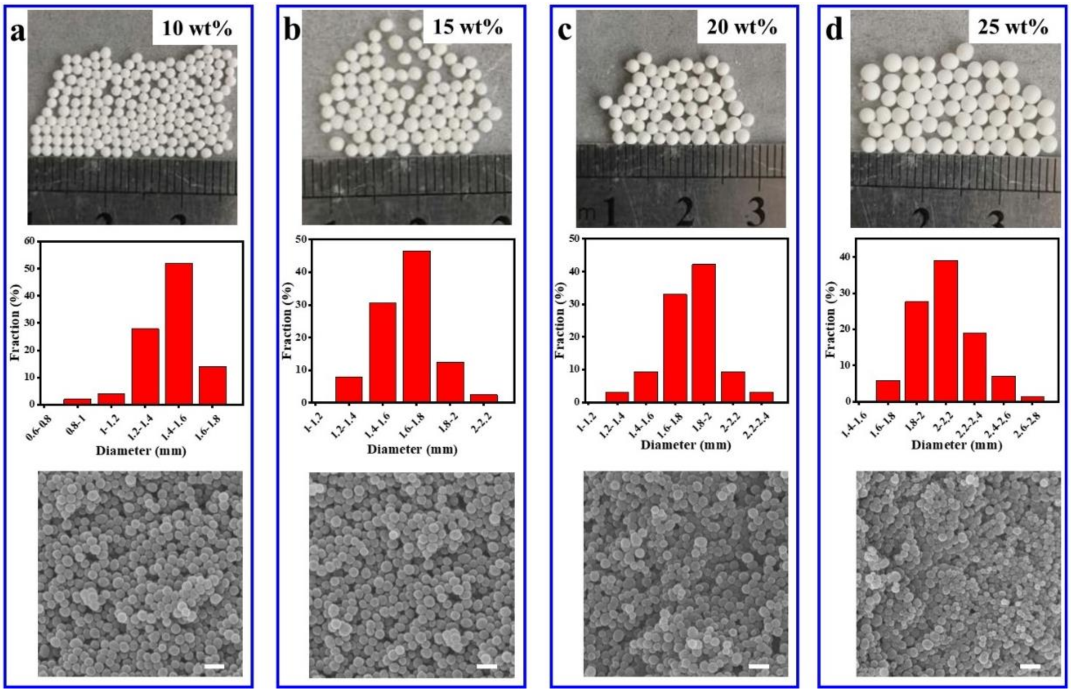

Supplementary Figure 18. Characterization of SPs prepared with different amounts of MSNs (the concentration of TEPA was fixed at 4 wt%). **a**, 10 wt%. **b**, 15 wt%. **c**, 20 wt%. **d**, 25 wt%. Scale bars = 200 nm.

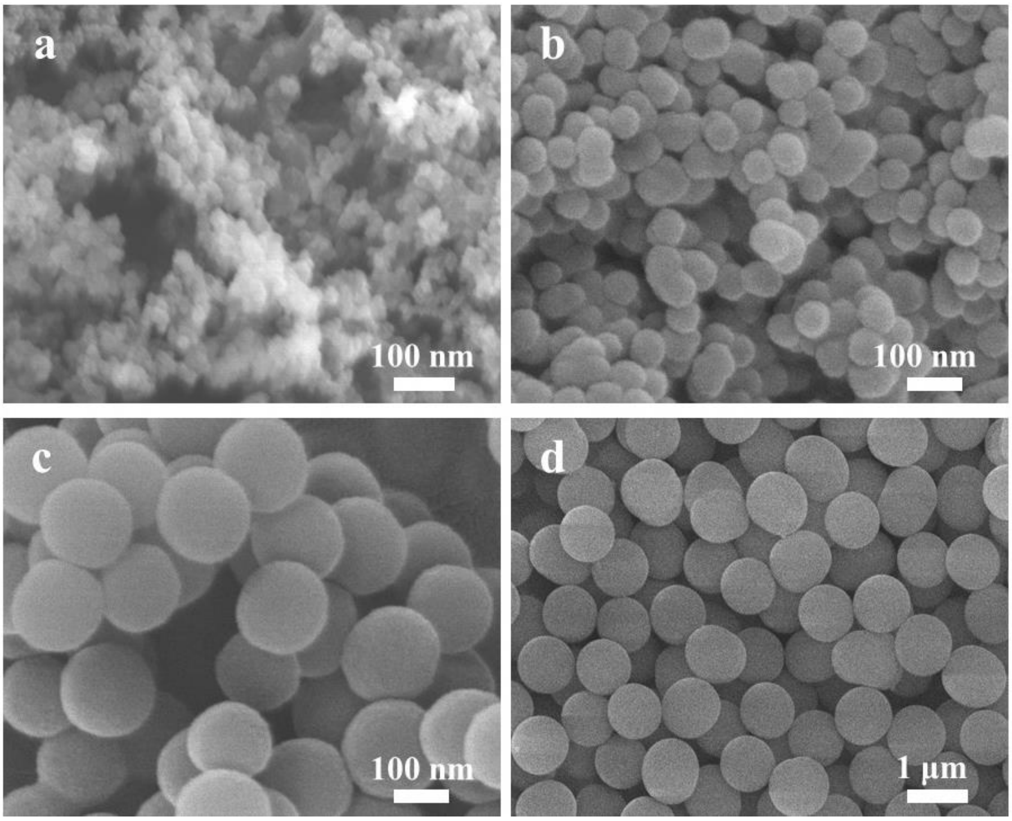

Supplementary Figure 19. SEM images of MSNs with different particle sizes. a, 20 nm. **b**, 60 nm. **c**, 180 nm. **d**, 880 nm.

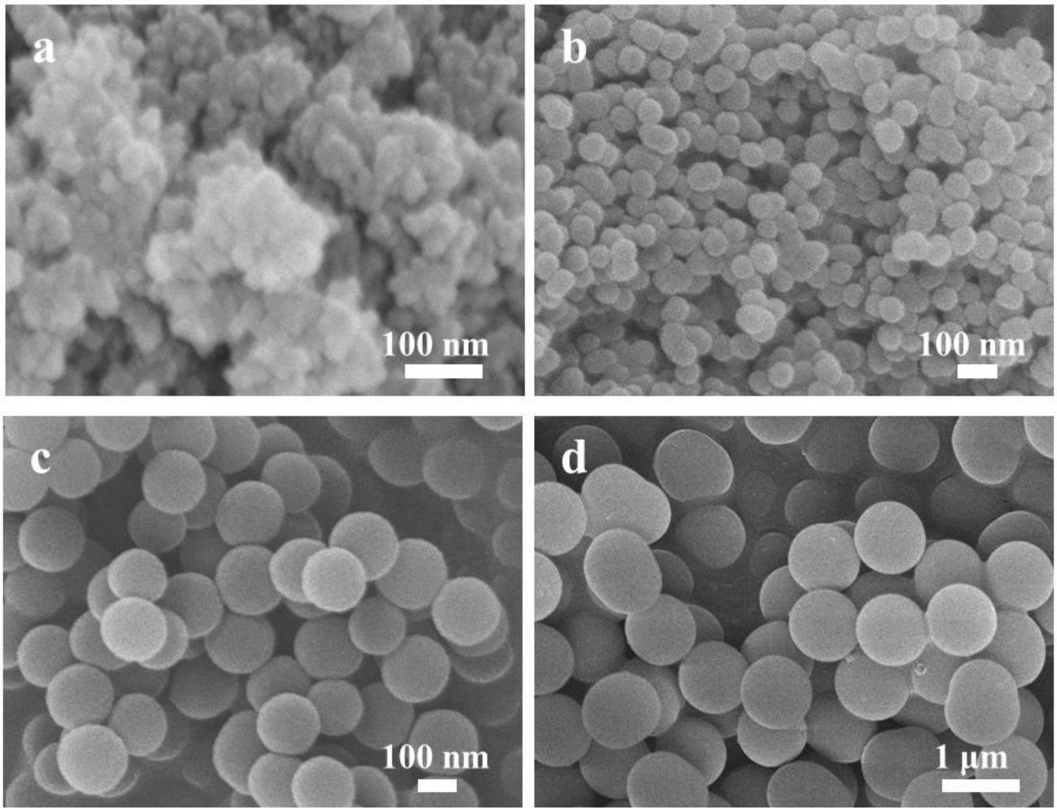

Supplementary Figure 20. SEM images showing the interior structure of SPs prepared with differently sized MSNs (keeping the amounts of TEPA and MSNs at 4 wt% and 10 wt%). **a**, 20 nm. **b**, 60 nm. **c**, 180 nm. **d**, 880 nm.

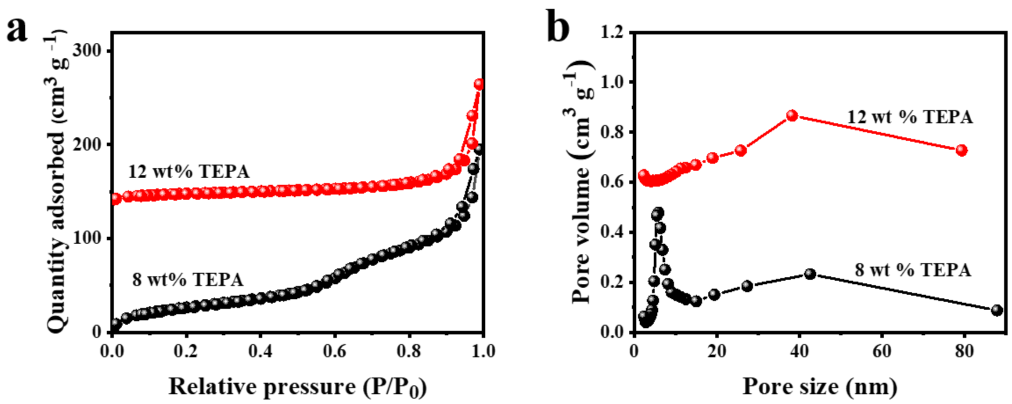

c Textural parameters of SPs prepared with different amounts of TEPA

Samples	S_{BET} (m^2 g^{-1})	V (cm^3 g^{-1})	Pore Size (nm)
8 wt% TEPA	102	0.30	5.1
12 wt% TEPA	30	0.19	—

Supplementary Figure 21. N_2 sorption measurement results of SPs prepared with different amounts of TEPA. a, N_2 adsorption-desorption isotherms, offset vertically the prior one by 140. **b**, BJH pore size distribution plots, offset vertically the prior one by 0.6. **c**, Textural parameters of SPs.

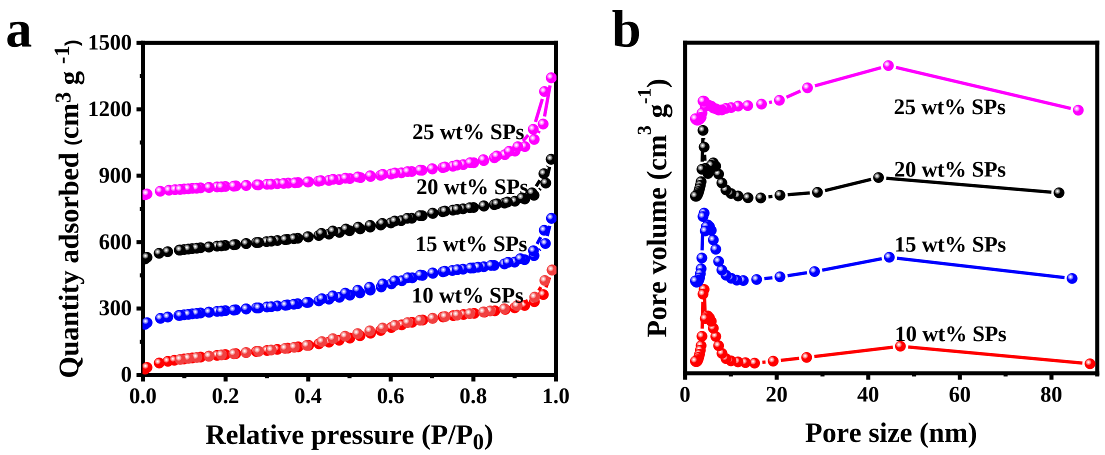

c Textural parameters of SPs prepared with different amounts of MSNs

Samples	S_{BET} (m^2 g^{-1})	V (cm^3 g^{-1})	Pore Size (nm)
10 wt%	347	0.73	3.9
15 wt%	341	0.78	3.9
20 wt%	322	0.73	3.9
25 wt%	198	0.83	3.9

Supplementary Figure 22. N_2 sorption measurement results of SPs prepared with different amounts of MSNs (the concentration of TEPA was fixed at 4 wt%). **a**, N_2 adsorption-desorption isotherms, offset vertically the prior one by 200, 500 and 800, respectively. **b**, BJH pore size distribution plots. **c**, Textural parameters of SPs.

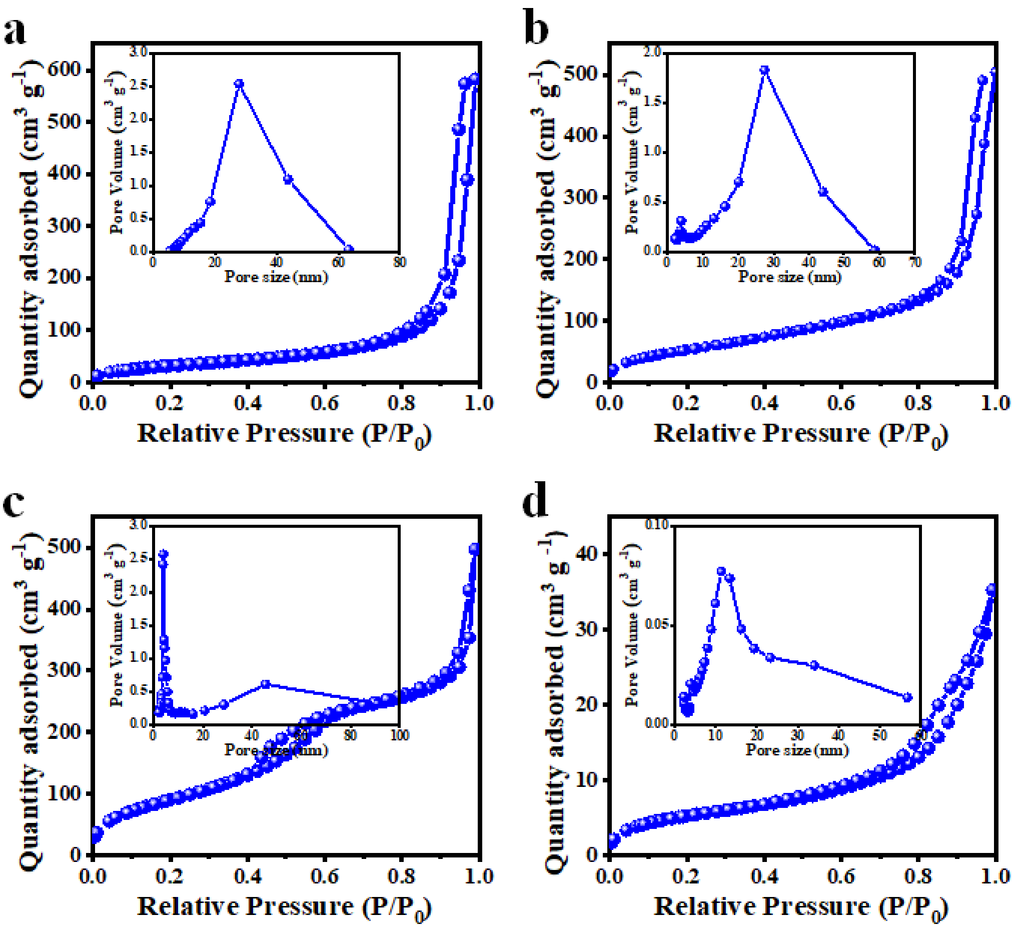

e Textural parameters of SPs prepared with different sizes of MSNs

Samples	S_{BET} (m^2 g^{-1})	V (cm^3 g^{-1})	Pore Size (nm)
20 nm	124	0.9	-
60 nm	201	0.78	3.9
180 nm	340	0.78	4.1
880 nm	20	0.05	-

Supplementary Figure 23. N_2 sorption measurement results of SPs prepared with differently sized MSNs. **a**, 20 nm. **b**, 60 nm. **c**, 180 nm. **d**, 880 nm. **e**, Textural parameters of different SPs.

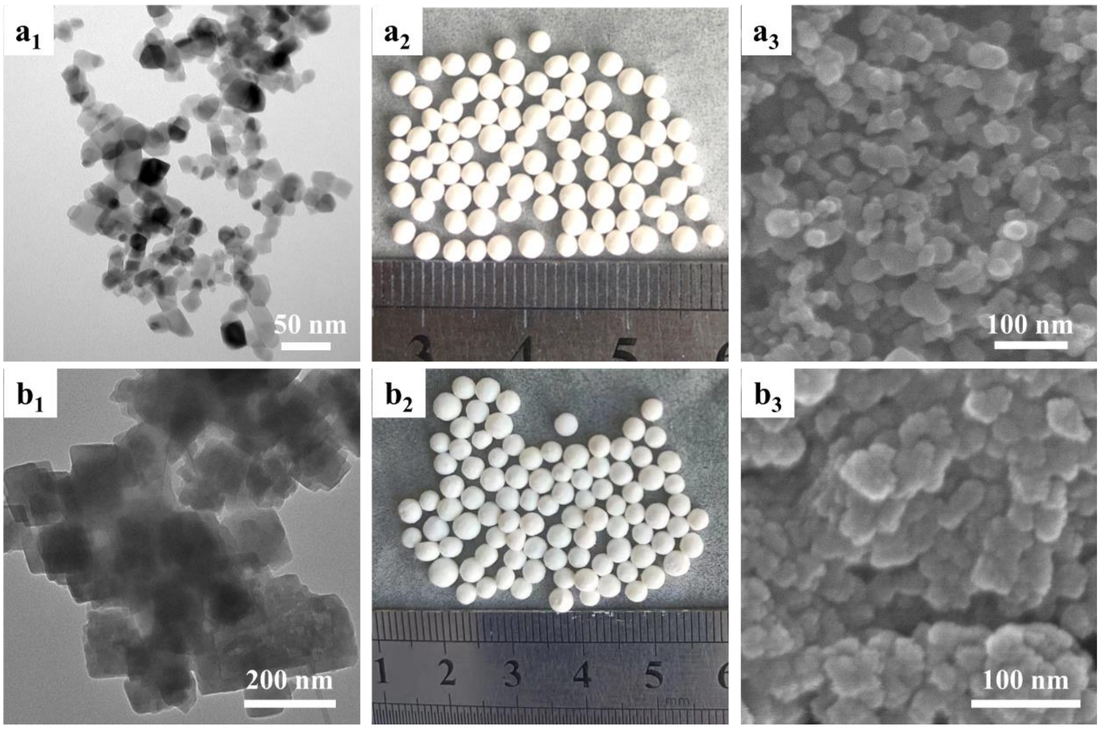

Supplementary Figure 24. Characterization of SPs prepared with different building blocks. **a1**, TEM image of nonporous TiO_2. **a2**, Appearance of SPs (here SPs were prepared 10 wt% TiO_2 and 4 wt% TEPA). **a3**, SEM image showing the interior structure of SPs prepared with nonporous TiO_2. **b1**, TEM image of H-beta zeolite. **b2**, Appearance of SPs (here SPs were prepared 10 wt% H-beta zeolite and 4 wt% TEPA). **b3**, SEM image showing the interior structure of SPs prepared with H-beta zeolite.

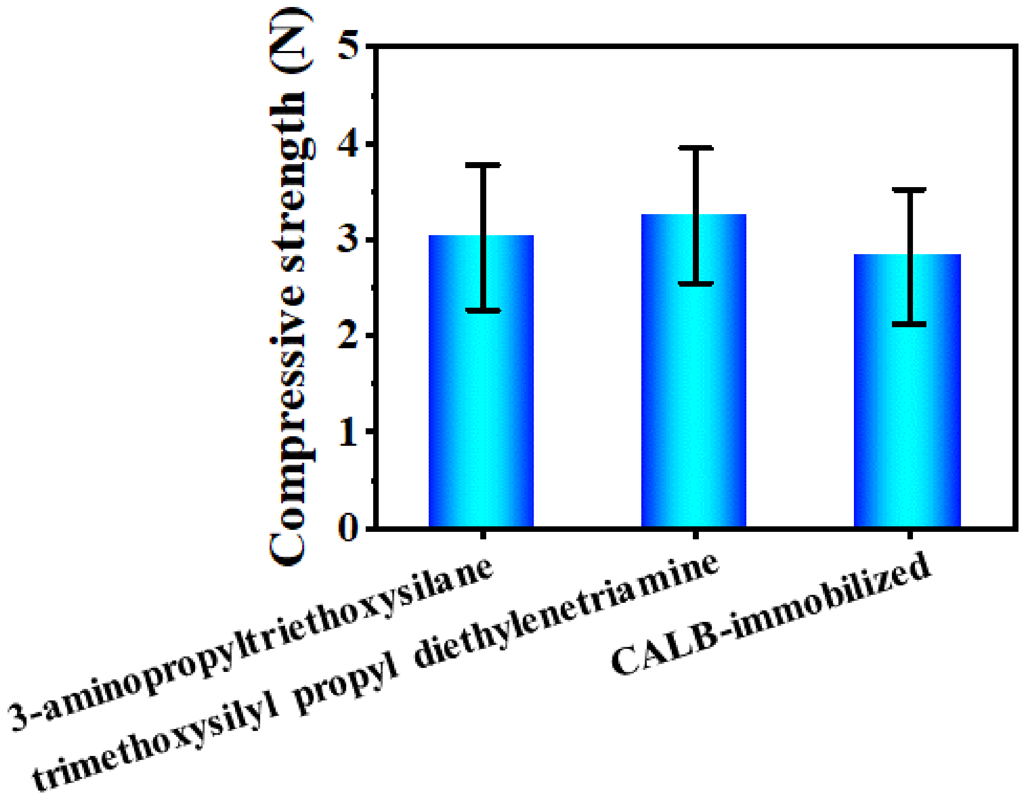

Supplementary Figure 25. Mechanical strength for SPs prepared with various organo-modified MSNs or CALB-immobilized MSNs. Error bars indicate the standard error from 20 samples averages.

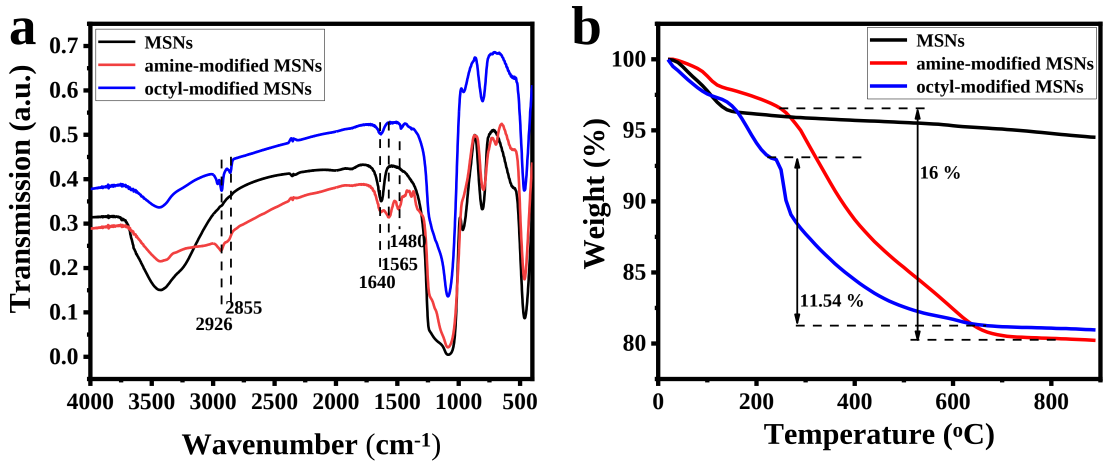

Supplementary Figure 26. Characterization of MSNs, amine-modified MSNs and octyl-modified MSNs. a, FT-IR spectra. **b**, TG curves.

Notes：According to TGA results, the loading of amine and octyl group on MSNs are estimated to be 0.91 mmol/g and 0.56 mmol/g, respectively.

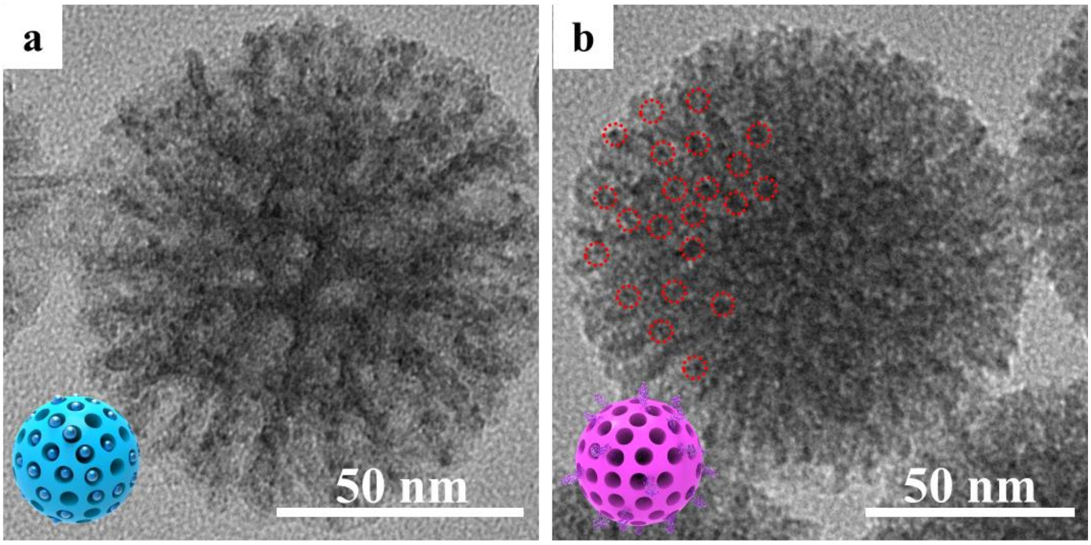

Supplementary Figure 27. Characterization of Pd/MSNs and CALB/MSNs. a, TEM image of Pd/MSNs. **b**, TEM image of CALB/MSNs, where CALB is labelled by Au clusters.

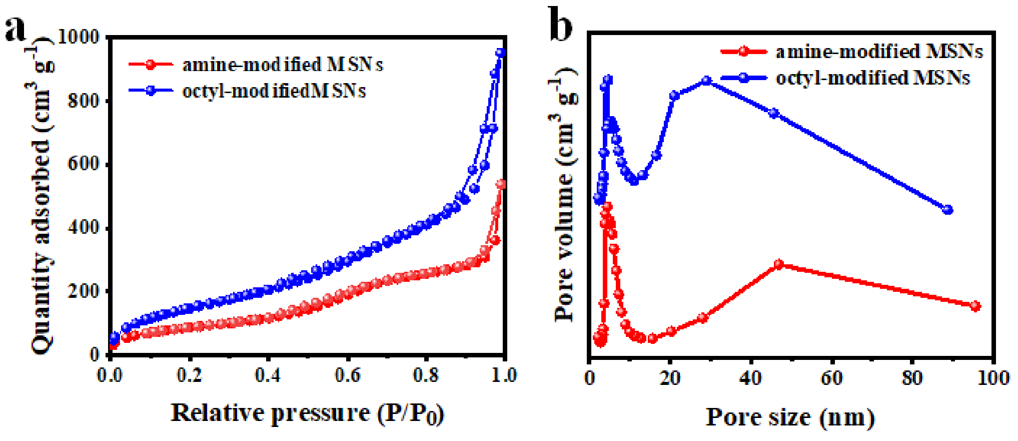

c Textural parameters of amine-modified MSNs and octyl-modified MSNs

Samples	S_{BET} (m^2 g^{-1})	V (cm^3 g^{-1})	Pore Size (nm)
amine-modified MSNs	327	0.84	4.5
octyl-modified MSNs	561	1.47	4.7

Supplementary Figure 28. N_2 sorption measurement results of amine-modified MSNs and octyl-modified MSNs. a, N_2 adsorption-desorption isotherms. **b**, BJH pore size distribution plots. **c**, Textural parameters of amine-modified MSNs and octyl-modified MSNs.

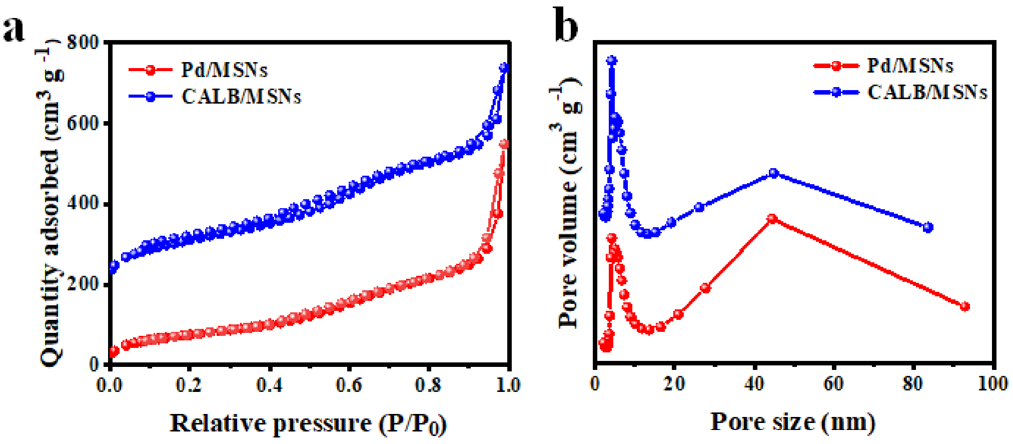

c Textural parameters of Pd/MSNs and CALB/MSNs

Samples	S_{BET} ($m^2\ g^{-1}$)	V ($cm^3\ g^{-1}$)	Pore Size (nm)
Pd/MSNs	281	0.85	4.2
CALB/MSNs	416	0.83	4.1

Supplementary Figure 29. N_2 sorption measurement results of Pd/MSNs and CALB/MSNs. a, N_2 adsorption-desorption isotherms, offset vertically the prior one by 200. **b**, BJH pore size distribution plots, offset vertically the prior one by 0.8. **c**, Textural parameters of Pd/MSNs and CALB/MSNs.

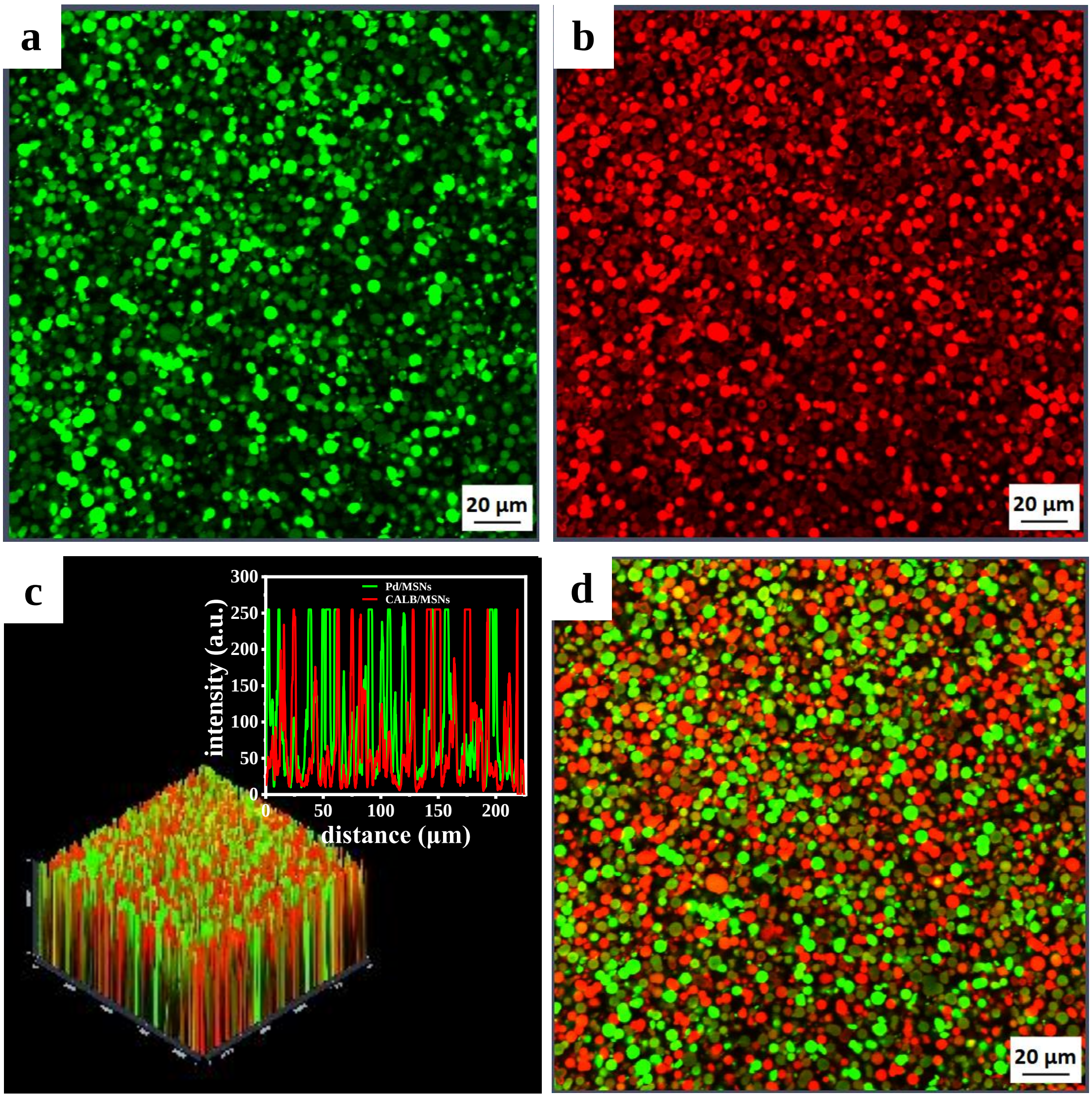

Supplementary Figure 30. Fluorescence confocal microscopy images of SPs prepared with MSNs (*ca.* 5 μm in diameter). **a**, Fluorescence confocal microscopy image of Pd-CALB/SPs with Pd/MSNs labelled by FITC-I. **b**, Fluorescence confocal microscopy image of Pd-CALB/SPs with CALB/MSNs labelled by Rhodamine B. **c**, 2.5D confocal laser scanning microscopy image of Pd-CALB/SPs with Pd/MSNs and CALB/MSNs labelled by FITC-I and Rhodamine B, respectively (the inset is fluorescence intensity profiles showing the distribution of Pd/MSNs and CALB/MSNs within the Pd-CALB/SPs). **d**, Fluorescence confocal microscopy image of Pd-CALB/SPs with Pd/MSNs and CALB/MSNs labelled by FITC-I and Rhodamine B, respectively.

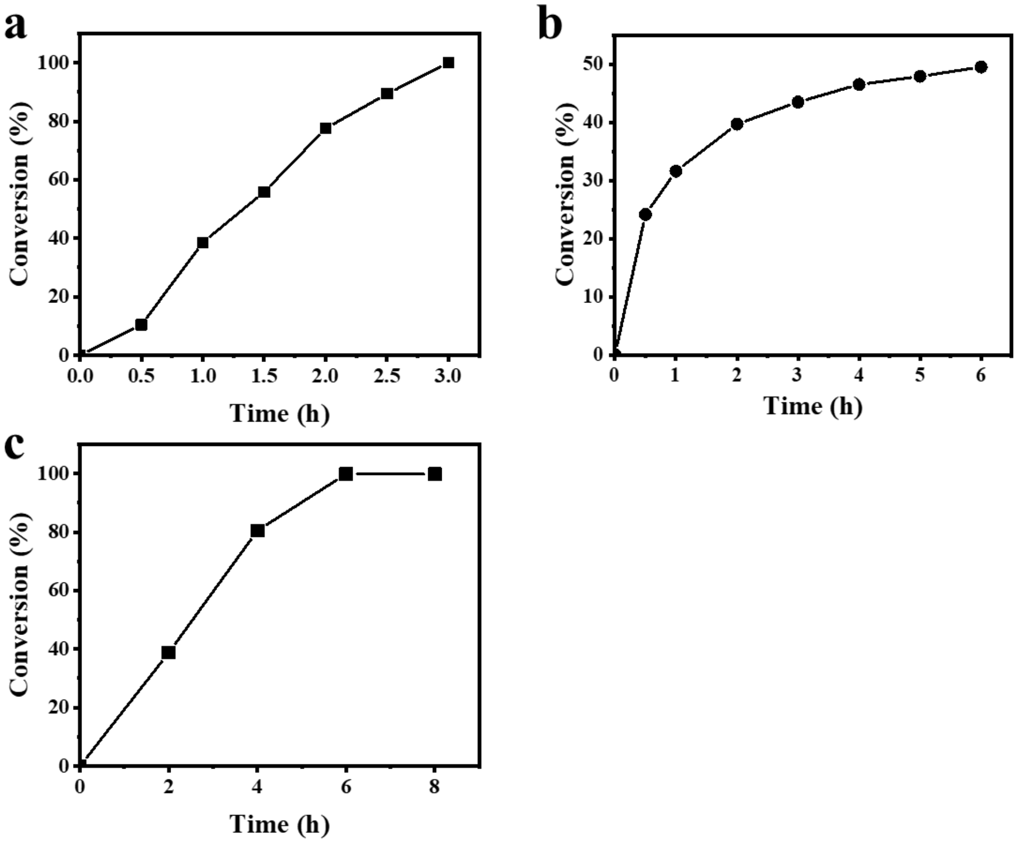

Supplementary Figure 31. Kinetic profiles of individual step and cascade reactions. **a**, Kinetic results for acetophenone hydrogenation over Pd/SPs, reaction condition: 0.2 mmol acetophenone, 2.0 mL *n*-octane, 2 MPa H_2, 50 °C and the molar ratio of acetophenone to Pd (S/C) = 56:1. **b**, Kinetic results for kinetic resolution of 1-phenylethanol over CALB/SPs, reaction condition: 0.2 mmol 1-phenylethanol, 0.8 mmol vinyl acetate, 2.0 mL *n*-octane, 50 °C. **c**, Kinetic results for sequential hydrogenation-kinetic resolution of acetophenone over Pd-CALB/SPs, 0.2 mmol acetophenone, 0.8 mmol vinyl acetate, 2.0 mL *n*-octane, 2 MPa H_2, 50 °C and the molar ratio of acetophenone to Pd (S/C) = 56:1.

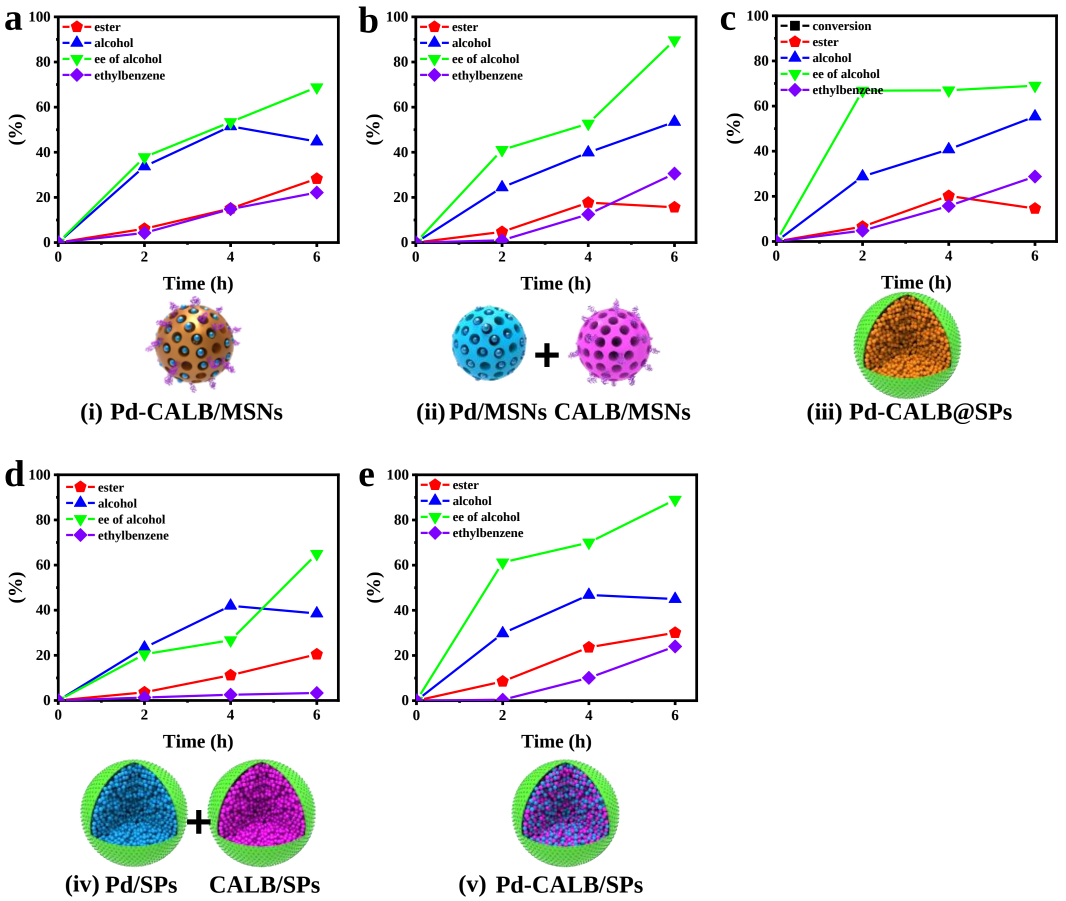

Supplementary Figure 32. Kinetic profiles of cascade of ketone hydrogenation and alcohols kinetic resolution over different catalysts in batch reactions. Reaction conditions: 60 mg catalyst, 0.20 mmol acetophenone, 0.80 mmol vinyl acetate, 2.0 mL *n*-octane, 2 MPa H_2, 50 °C. **a**, 30 mg Pd-CALB/MSNs. **b**, A physical mixture of 30 mg Pd/MSNs and 30 mg CALB/MSNs. **c**, 30 mg Pd-CALB@SPs. **d**, A physical mixture of 30 mg Pd/SPs and 30 mg CALB/SPs. **e**, 60 mg Pd-CALB/SPs.

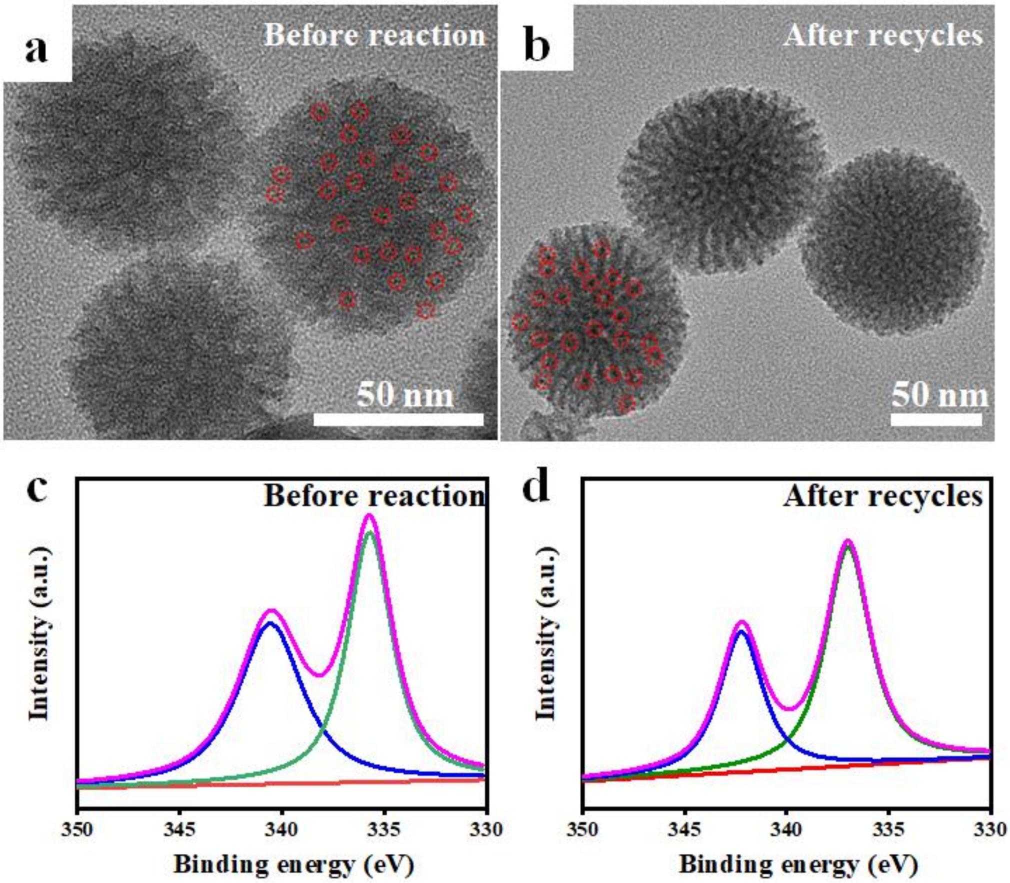

Supplementary Figure 33. Characterization of Pd-CALB@SPs. **a**, TEM image showing the interior structure of fresh Pd-CALB@SPs (Pd NPs and CALB were co-localized on a single MSN particle). **b**, TEM image showing the interior structure of Pd-CALB@SPs after four reaction cycles. **c**, Pd 3*d* XPS spectra of fresh Pd-CALB@SPs. **d**, Pd 3*d* XPS spectra of Pd-CALB@SPs after four reaction recycles.

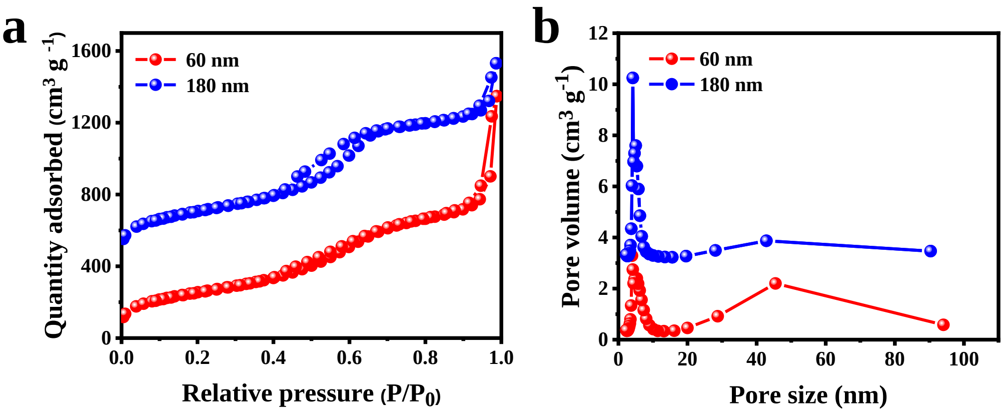

c Textural parameters of Pd-CALB/SPs prepared with differently sized MSNs

Samples	S_{BET} (m^2 g^{-1})	V (cm^3 g^{-1})	Pore Size (nm)
60 nm	241	0.63	5.1
180 nm	240	0.40	4.6

Supplementary Figure 34. N_2 sorption measurement results of Pd-CALB/SPs prepared with differently sized MSNs. a, N_2 adsorption-desorption isotherms, offset vertically the prior one by 200. **b**, BJH pore size distribution plots, offset vertically the prior one by 0.6. **c**, Textural parameters of Pd-CALB/SPs.

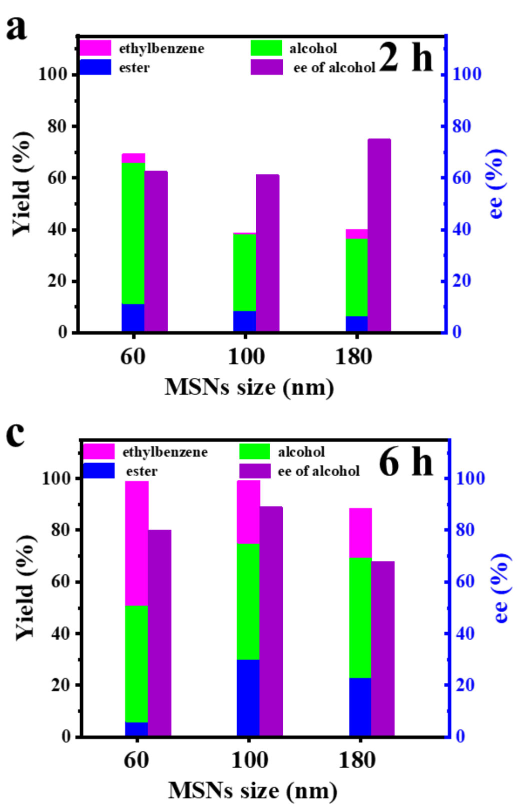

Supplementary Figure 35. Reaction results of sequential hydrogenation-kinetic resolution of acetophenone over SPs prepared with differently sized MSNs (at different reaction times). Reaction conditions: 60 mg catalyst, 0.20 mmol acetophenone, 0.80 mmol vinyl acetate, 2.0 mL *n*-octane, 2 MPa H_2, 50 ºC. **a**, 2 h. **b**, 4 h. **c**, 6 h.

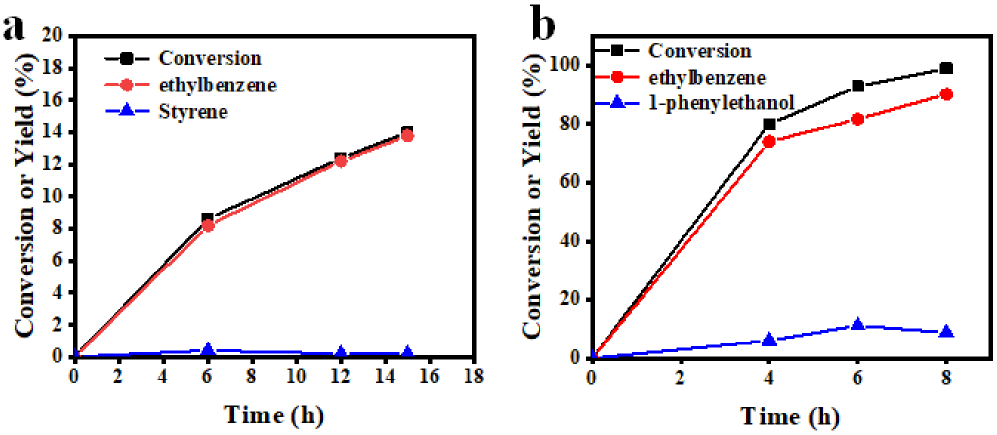

Supplementary Figure 36. Kinetic profiles of 1-phenylethanol hydrogenation and 1-phenylethyl acetate hydrogenation over Pd-CALB/SPs. Reaction condition: 60 mg catalyst, 2.0 mL *n*-octane, 2 MPa H_2, 50 °C. **a**, 0.2 mmol 1-phenylethanol. **b**, 0.2 mmol 1-phenylethyl acetate.

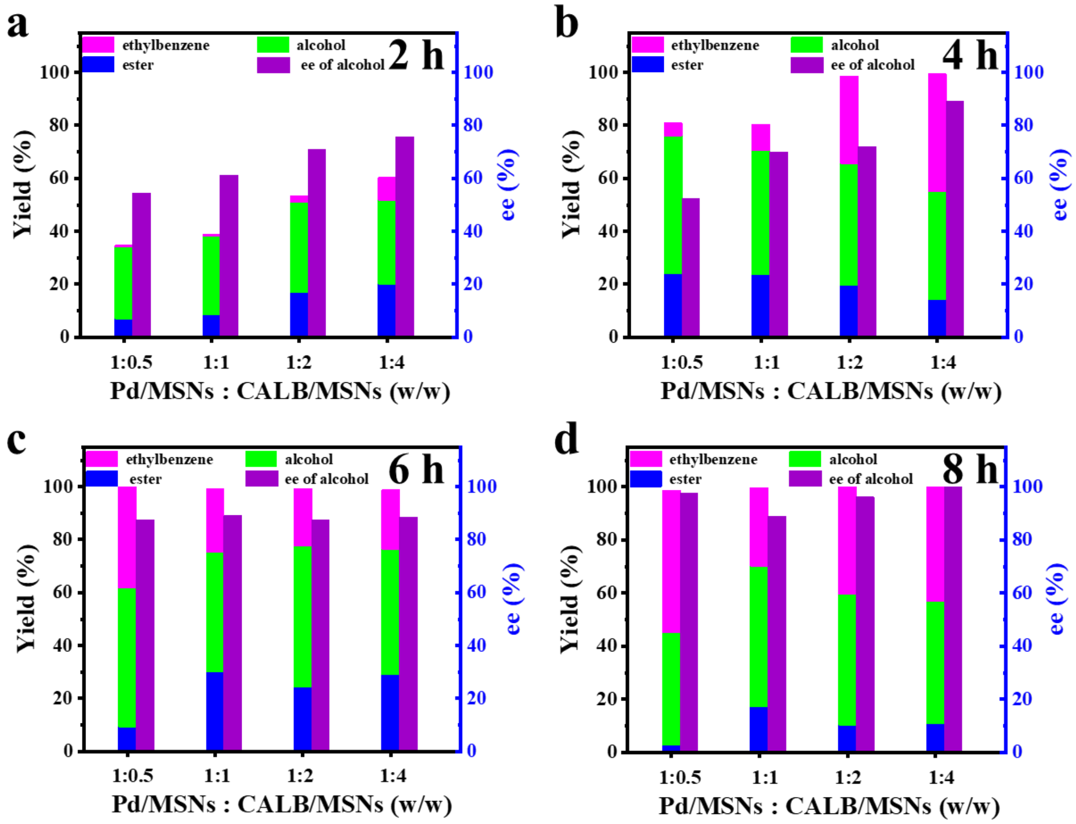

Supplementary Figure 37. Results of sequential hydrogenation-kinetic resolution of acetophenone over SPs prepared with different mass ratios of Pd/MSNs to CALB/MSNs (at different reaction time). Reaction conditions: 0.20 mmol acetophenone, 0.80 mmol vinyl acetate, 2 mL *n*-octane, 2 MPa H_2, 50 °C, 30 mg Pd/MSNs, 15 mg, 30 mg, 60 mg or 120 mg CALB/MSNs. **a**, 2 h. **b**, 4 h. **c**, 6 h. **d**, 8 h.

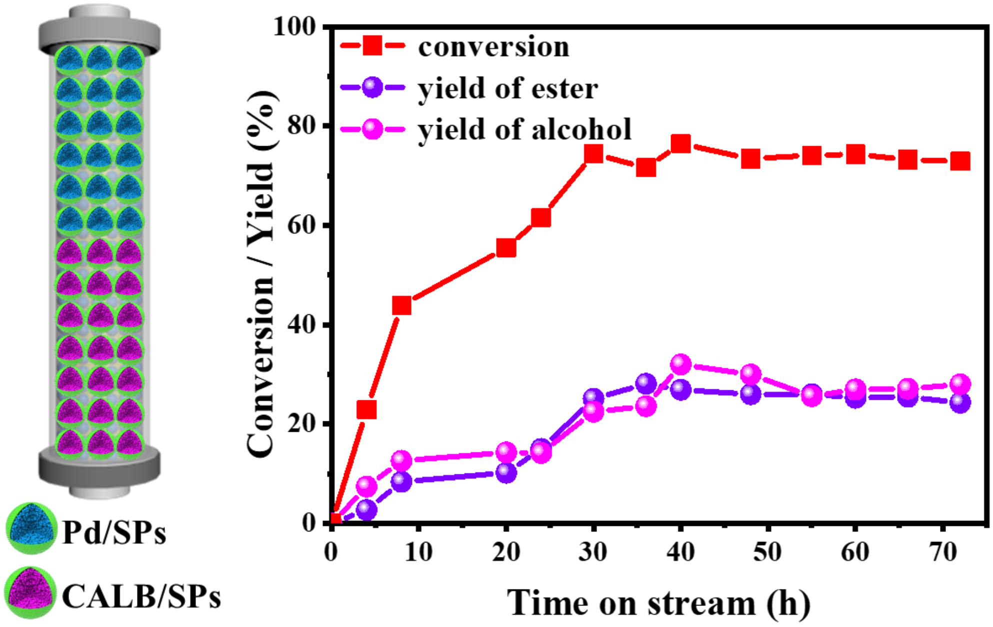

Supplementary Figure 38. Results of the cascade of hydrogenation and kinetic resolution over Pd/SPs and CALB/SPs in two-stage reactions. Reaction conditions: 0.1 M acetophenone, 0.4 M vinyl acetate in *n*-octane, 0.425 g Pd/SPs, 0.425 g CALB/SPs, 50 °C, 2 MPa H_2, flow rate from initial 3.0 mL h^{-1} to 1.5 mL h^{-1} at the end.

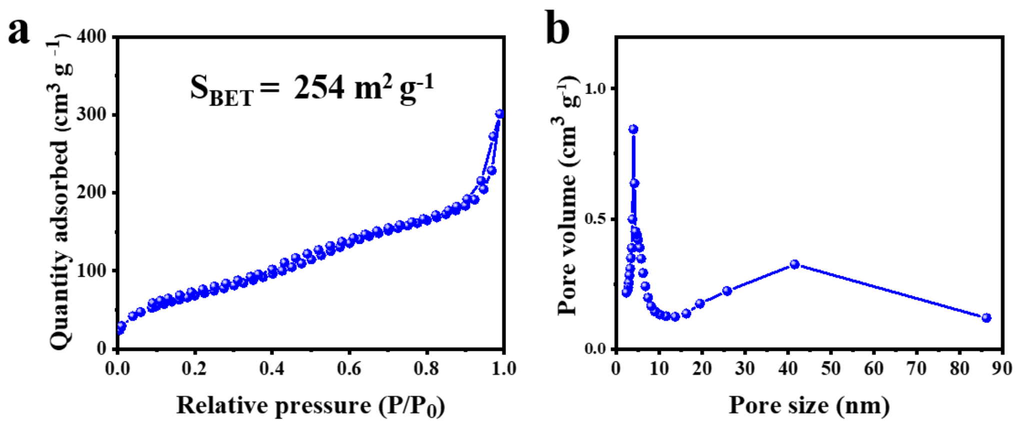

Supplementary Figure 39. N_2 sorption measurements results of Pd-CALB/SPs after 200 h of continuous flow reaction. **a**, N_2 adsorption-desorption isotherm. **b**, BJH pore size distribution.

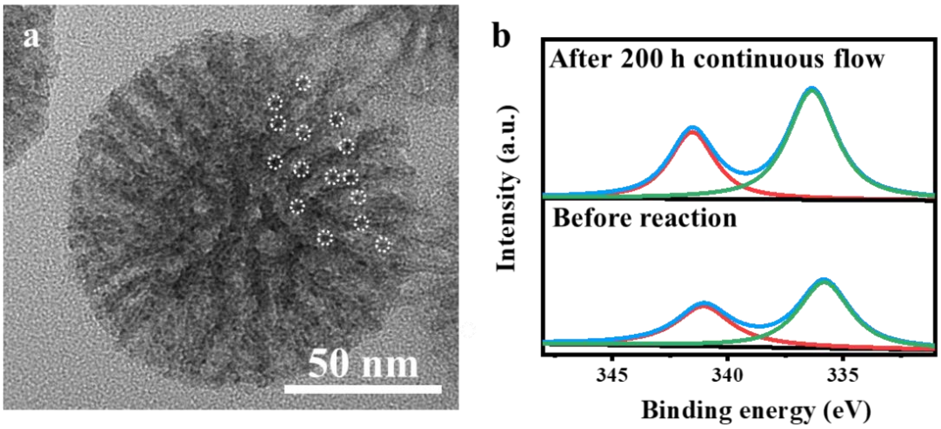

Supplementary Figure 40. Characterization of Pd-CALB/SPs after 200 h of continuous flow reaction. a, TEM image of Pd/MSNs in Pd-CALB/SPs after 200 h of continuous flow reaction. **b**, Pd 3*d* XPS spectra of Pd-CALB/SPs before and after 200 h of continuous flow reaction.

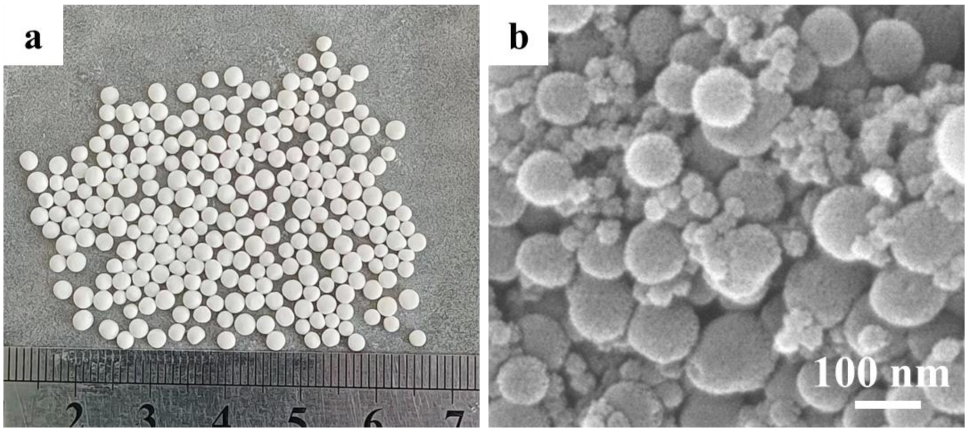

Supplementary Figure 41. Characterization of H-beta-CALB/SPs. **a**, Appearance of H-beta-CALB/SPs. **b**, SEM image showing the interior structure of H-beta-CALB/SPs.

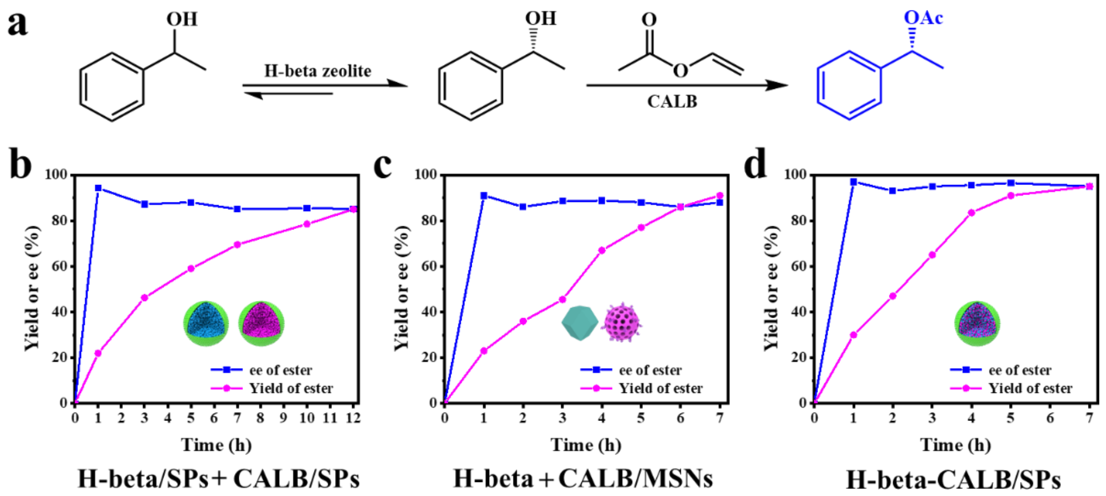

Supplementary Figure 42. Results of the cascade reaction for the synthesis of chiral 1-phenylethanol acetate over different catalysts. a, Reaction network of the cascade reaction. **b**, Kinetic profiles of the cascade reaction over a physical mixture of 30 mg H-beta/SPs and 30 mg CALB/SPs (CALB loading, 46.5 mg g^{-1}), reaction conditions: 3 mL *n*-octane containing 1-phenylethanol (0.1 M) and vinyl acetate (0.4 M), 45 °C, 600 rpm. **c**, Kinetic profiles of the cascade reaction over a physical mixture of 30 mg H-beta zeolite and 30 mg CALB/MSNs (CALB loading, 46.5 mg g^{-1}), reaction conditions are the same as in (**b**). **d**, Kinetic profiles of the cascade reaction over 60 mg H-beta-CALB/SPs (CALB loading, 23.3 mg g^{-1}), reaction conditions are the same as in (**b**).

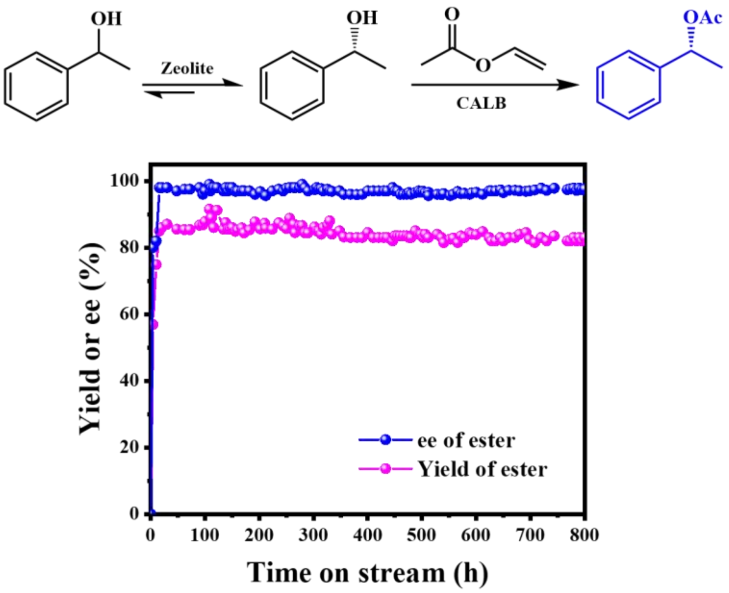

Supplementary Figure 43. Results of the cascade reaction for the synthesis of chiral 1-phenylethanol acetate over H-beta-CALB/SPs in a continuous flow system. Reaction condition: 1 g H-beta-CALB/SPs packed in a fixed-bed reactor, 0.1 M 1-phenylethanol and 0.4 M vinyl acetate in *n*-octane, 45 °C, flow rate at 2.5 mL h^{-1} from 0 to 300 h, flow rate at 2.0 mL h^{-1} from 300 to 450 h and 1.5 mL h^{-1} after 450 h.

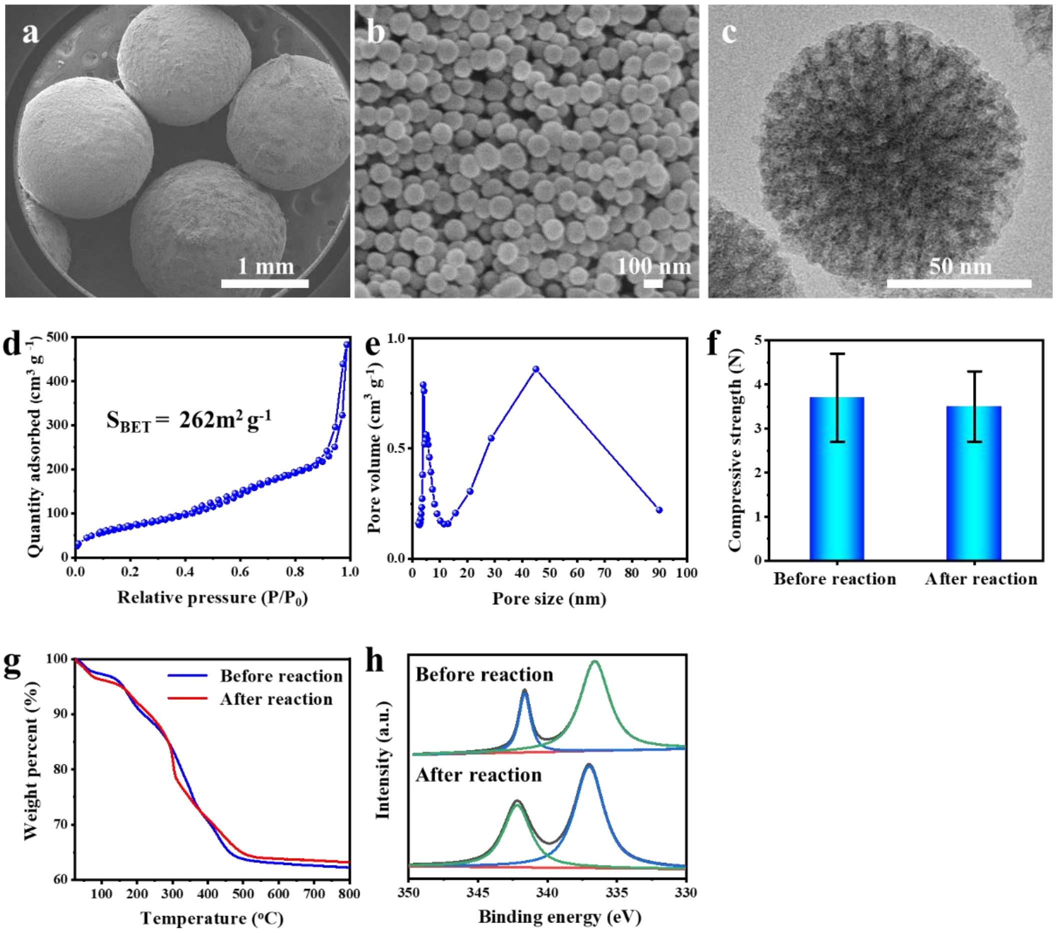

Supplementary Figure 44. Characterization of Pd-CALB/SPs after 500 h of continuous flow reaction. a, SEM image of Pd-CALB/SPs after 500 h of continuous flow reaction. **b**, SEM image showing the interior structure of Pd-CALB/SPs after 500 h of continuous flow reaction. **c**, TEM image of Pd-CALB/SPs after 500 h of continuous flow reaction. **d**, N_2 adsorption-desorption isotherm of Pd-CALB/SPs after 500 h of continuous flow reaction. **e**, BJH pore size distribution plot of Pd-CALB/SPs after 500 h of continuous flow reaction. **f**, Mechanical strength for Pd-CALB/SPs before and after 500 h of continuous flow reaction. Error bars indicate the standard error from 20 samples averages. **g**, TG curves of Pd-CALB/SPs after 500 h of continuous flow reaction. **h**, Pd 3*d* XPS spectra of Pd-CALB/SPs before and after 500 h of continuous flow reaction.

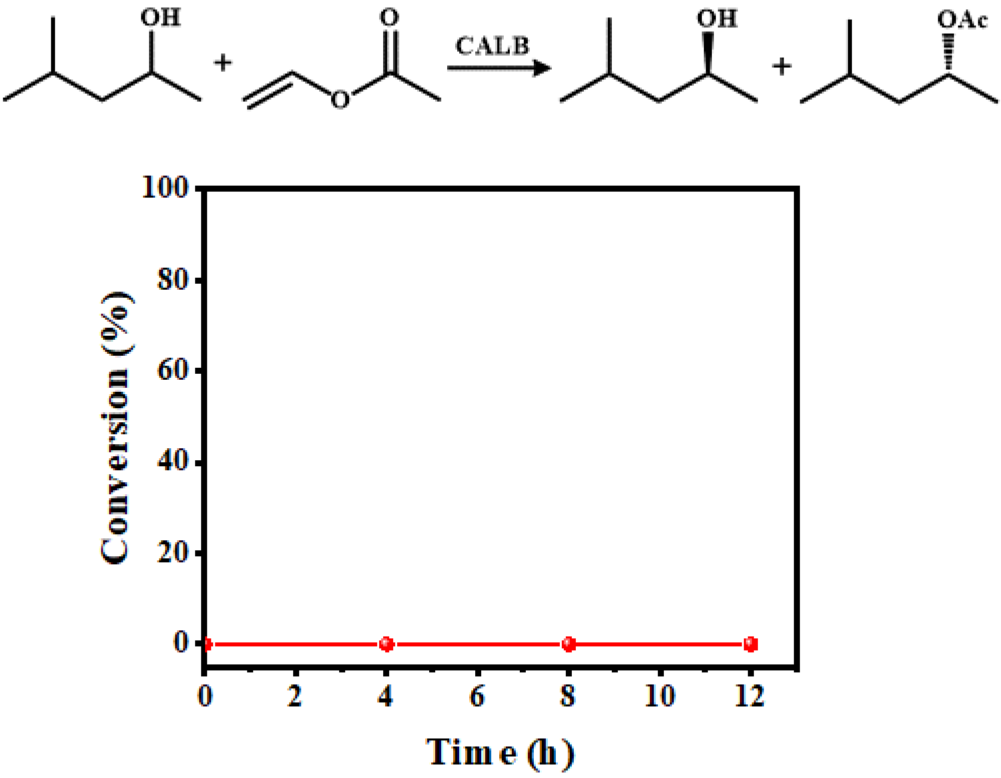

Supplementary Figure 45. Results of testing CALB activity in the eluent from sequential amine racemization-kinetic resolution. Reaction condition: 0.1 mmol 4-methyl-2-pentanol, 0.4 mmol vinyl acetate and 10 mL outflow from the fixed-bed reaction (amine racemization and kinetic resolution over Pd-CALB/SPs), 50 °C.

Notes: To check whether the flow process leads to the leaching of CALB from Pd-CALB/SPs, we examined the catalytic activity of the outflow by adding another substrates (4-methyl-2-pentanol) instead of previous 1-phenylamine. As shown above, no new products were determined even during 12 h of reaction, indicating no significant CALB leaching from the fixed-bed reactor. Moreover, a protein assay was also performed to further examine the CALB leaching. The result was determined to be below the detection limit.

Supplementary Table 1. Results of ICP-MS analysis of Pd-CALB/SPs before and after 200 h of continuous flow reaction

sample	Pd (wt%)	P (wt%)
Pd-CALB/SPs	0.63	0.12
Pd-CALB/SPs after 200 h continuous flow	0.54	0.10

Supplementary Table 2. Results of ICP-MS analysis of Pd-CALB@SPs before and after four reaction recycles

sample	Pd (wt%)	P (wt%)
Before reaction	1.25	0.36
After four reaction recycles	1.00	0.33

Supplementary Table 3. Loadings of Pd and CALB in various catalysts

sample	Pd (wt%)	CALB (mg g^{-1})
Pd-CALB/MSNs	1.25	47.3
Pd/MSNs	1.26	
CALB/MSNs		46.5
Pd-CALB@SPs	1.25	47.3
Pd/SPs	1.26	
CALB/SPs		46.5
Pd-CALB/SPs	0.63	23.3

Supplementary Table 4. Results of ICP-MS analysis of Pd-CALB/SPs before and after 500 h of continuous flow reaction

sample	Pd (wt%)	P (wt%)
Before reaction	0.65	0.125
After 500 h continuous-flow	0.55	0.09

Supplementary Methods

1. Chemicals

Toluene (AR), *n*-octane (AR), dimethyl sulfoxide (AR), cyclohexane (AR), p-xylene (AR), ethyl acetate (AR), sodium dihydrogen phosphate (NaH_2PO_4) and dibasic sodium phosphate (Na_2HPO_4) were procured from Sinopharm Chemical Reagent Co., LTD, (China). Concentrated hydrochloric acid (37 wt%) was bought from QiXian Hui Hong Yuan Chemical Co., LTD Company. Native Lipase B from *Candida Antarctica* (CALB) was purchased from Novozymes. Pluronic P123 ($EO_{20}PO_{70}EO_{20}$, Mv~5800), ammonium fluoride (NH_4F, AR), tetraethyl orthosilicate (TEOS, AR), *n*-hexylamine (99%), (octyl)-trimethoxysilane (97%), hexadecyltrimethylammonium chloride (CTAC, 97%), triethanolamine (TEA, AR), 3-aminopropyltriethoxysilane (APTES, 99%), sodium tetrachloropalladate (Na_2PdCl_4, 98%), sodium borohydride ($NaBH_4$, 98%), acetophenone (98%), 4'-methylacetophenone (98%), vinyl acetate (99%), ethyl methoxyacetate (98%), 1-phenylethylamine (98%), 1-(4-methylphenyl)ethylamine (97%), 1-(4-fluorophenyl)ethylamine (97%), 1,2,3,4-tetrahydro-1-naphthylamine (98%), 1-(4-methylphenyl)ethylamine (96%), dodecane (GC, >99%), hexadecane (98%), ethylene glycol (AR), ethanolamine (99.7%), ethylenediamine (AR), polyethylene glycol (M.W. 400), diethylenetriamine (97%), tetraethylenepentamine (TEPA), pentaethylenehexamine polyethyleneimine (PEI, M.W. 800, 98%), fluorescein isothiocyanate isomer I (FITC-I, CAS No. 3326-32-7) and Rhodamine B (CAS No. 81-88-9) were purchased from Aladdin (China). Chitosan (M.W. 800-1000), epoxy resin (Viscosity: 1100-1600 mpa.s), carboxymethylcellulose (CMC, M.W. 250000) were purchased from Macklin Biochemical Co., Ltd, (China). Nile red (CAS No. 7385-67-3) was purchased from Sigma-Aldrich. H-beta zeolite was purchased from Nankai University Catalyst Co., Ltd, (China). TiO_2 (P25) was purchased from Degussa. Water used in this study was deionized water.

2. Characterization

Transmission electron microscopy (TEM) images were obtained on a JEM 2000EX (operated at 200 kV). Samples for TEM observations were prepared by dispersing the

sample powder in ethanol using ultrasound and then allowing a drop of the suspension to evaporate on a copper gird covered with a holey carbon film. Scanning electron microscope (SEM) images were obtained on a JEOL JSM-6700F field-emission electron microscope. EDS elemental maps were recorded on this instrument. Nitrogen-sorption measurements were performed at −196 °C on a Micromeritics ASAP 2020 analyzer. Before measurement, all samples were out gassed at 150 °C under vacuum for 6 h. The specific surface area was calculated from the adsorption branch in the relative pressure range of 0.05-0.15 using the Brunauer-Emmett-Teller (BET) method. X-ray photoelectron spectroscopy (XPS) was performed under an ultrahigh vacuum on a Kratos AXIS ULTRA DLD spectrometer with Al Ka radiation and a multichannel detector. Fourier transform infrared spectroscopy (FT-IR) spectra were recorded on a Nicolet Nexus 470 FT-IR spectrometer over the range 4000−400 cm^{-1} with 64 scans and a resolution of 2 cm^{-1}. The samples were performed under vacuum with a heating rate of 5 °C min^{-1} to 150 °C for 2 h in vacuum, then recorded the spectra. Solid-state ^{13}C-MAS NMR measurements were performed on Varian Infinity Plus 400 NMR spectrometer. The spinning rate was 4 kHz and a total number of 800 scans were recorded with 4 s recycle delay for each sample. Solid-state ^{1}H CP-MAS NMR measurements were performed on a Bruker AVANCE IIITM 600 MHz spectrometer. The spectra were recorded by hahn-echo method at an MAS rate of 12 kHz. The samples dehydration were carried out on glove box with 150 °C for 10 h, then packed on a zirconia rotor. Thermogravimetric (TG) measurement was performed on TA Q-600 instrument. UV-Vis determination was conducted on a TU-1900 spectrometer (China). Gas chromatography (GC) analysis was carried out on an Agilent 7890 analyzer (Agilent-19091G-B213, HP-CHIRAL-20B, CYCLODEX-B) with a flame ionization detector. The identification of products by mass spectrometry (MS) was performed on a GC-MS instrument (7890B-5977A, HP-5, Agilent). Confocal laser scanning microscopy (CLSM) images were obtained on a Carl Zeiss LSM880 instrument (Germany). The excitation wavelength of FITC-I is 488 nm (green). The excitation wavelength of Nile Red is 559 nm (red). The concentration of Rhodamine B in a

solution (a mixture of ethanol and water, 1:1 V/V) was 2×10^{-6} M and the excitation wavelength is 554 nm (red). The piston pump (LC-01P) used in this study was purchased from Jiangshen Technology Co., China. Viscosity measurements were performed at 25 °C on Brookfield DV-Ⅱ+ Pro analyzer.

3. Material synthesis

Preparation of spongy porous silica. Mesoporous silica nanoparticles were prepared according to the reported method[1]. Typically, 4.8 g P123 was dissolved in 168 mL HCl solution (1.30 M). Then, the resultant suspension was stirred at 40 °C until the solution became clear, followed by addition of 0.054 g NH_4F. After stirring at this temperature for 10 min, a mixture of 21.64 g *n*-octane and 8.1 g TEOS was then added into the above solution. The resulting mixture was keep stirred at 40 °C for 24 h and then transferred into an autoclave for further condensation at 100 °C for 48 h. The resultant solid was collected by filtration, then dried in air and calcined at 550 °C for 5 h, eventually leading to the spongy porous silica.

Preparation of hydrophobic spongy porous silica. 1.0 g as-synthesized spongy porous silica (dried at 120 °C for 4 h) were dispersed into 50 mL *p*-xylene using sonication for 20 min. Then, 4 mmol (octyl)-trimethoxysilane and 4 mmol *n*-hexylamine were added into this suspension. This mixture was stirred under a N_2 atmosphere at 130 °C for 10 h. The solid particles were collected through centrifugation, washed with toluene and dried, yielding octyl-modified spongy porous silica.

Preparation of mesoporous silica nanospheres (MSNs). MSNs were prepared according to a previously reported method[2]. 0.36 g TEA was added into 112 mL CTAC aqueous solution (10 wt%). The resultant mixture was gently stirred at 60 °C for 1 h. Then, a solution of TEOS in cyclohexane (20 v/v%, 40 mL) was slowly added to the above suspension, which was maintained at 60 °C in a water bath for 12 h under magnetic stirring. The solid product was collected by centrifugation and washed for several times with ethanol. After calcination in air at 550 °C for 5 h (ramping rate, 2 °C min^{-1}), MSNs were obtained. To tune the sizes MSNs (60 or 180 nm), the temperature

for hydrolysis-condensation of TEOS was decreased down to 45 °C or increased up to 75 °C).

Preparation of other SPs. The MSNs was replaced by TiO_2 or microporous H-beta zeolite. Other procedures are the same as the synthesis of SPs made of silica nanoparticles.

Preparation of amine-modified MSNs. 1.0 g MSNs (dried at 120 °C for 4 h) were dispersed into 50 mL toluene. Then 1 mL APTES was added into this suspension. This mixture was stirred under N_2 atmosphere at 110 °C for 4 h. The solid particles were collected through centrifugation, and washed with toluene and dried, yielding amine-modified MSNs.

Preparation of octyl-modified MSNs. 1.0 g MSNs (dried at 120 °C for 4 h) were dispersed into 50 mL toluene. Then, 0.234 g (octyl)-trimethoxysilane and 0.101 g *n*-hexylamine were added into this suspension. The solid particles were collected through centrifugation, and washed with toluene and then dried, eventually yielding octyl-modified MSNs.

Preparation of CALB-immobilized MSNs (CALB/MSNs). 0.5 g octyl-functionalized MSNs were added into 45 mL enzyme solution consisting of 7.5 mL crude CALB (8.0 mg mL^{-1} of protein) and 37.5 mL phosphate buffer solution (PBS: 0.05 M Na_2HPO_4-0.05 M NaH_2PO_4, pH = 7.4). To increase the dispersion of MSNs, 5 mL ethanol was also added this mixture. After slowly rotating at 25 °C for 12 h, the solid material was isolated by filtration, and washed three times with PBS (pH = 7.4, 0.05 M). After that, the obtained material was filtered out and dried under vacuum. The obtained material is denoted as CALB/MSNs. The loading of CALB on the solid materials was calculated on the basis of the difference in the CALB concentration in the PBS between before and after adsorption (Bradford method).

Preparation of Pd NPs-supported MSNs (Pd/MSNs). Pd nanoparticles (NPs) were supported on the amine-modified MSNs according to reported procedure[3]. 0.2 g amine-modified MSNs was dispersed into 50 mL of deionized water via sonication, leading to

a suspension. After this suspension was stirred for 10 min at 30 ℃, 1.88 mL of fresh Na_2PdCl_4 aqueous solution (20 mM) was added dropwise. After stirring at 30 ℃ for 4 h, 3.0 mL of 0.1 M $NaBH_4$ solution was added. The resultant mixture was aged at this temperature for 20 h. The resultant solid was collected by centrifugation, washed with water and dried, yielding Pd/MSNs.

Preparation of Au clusters labelled CALB. Au clusters-labeled CALB were prepared according to the reported procedure[4]. In a typical synthesis, 2.0 mL of aqueous $HAuCl_4$ solution (2.5×10^{-3} M) was added to 9 mL of CALB solution (consisting of 1.5 mL crude CALB and 7.5 mL PBS, pH = 7.4, 0.05 M). The mixture was mixed by stirring for 5 min. Afterward, a few droplets of aqueous NaOH solution (0.1 M) were added to this mixture to adjust the pH to ∼11. Then, the resultant mixture was heated to 90 °C under gentle stirring for 24 h, leading to an orange aqueous suspension. The solid was isolated from this suspension through centrifugation and then washed several times with PBS, eventually leading to Au clusters labeled CALB. The immobilization procedure of the Au clusters labeled CALB composites on MSNs was the same as the immobilization of CALB on MSNs.

Preparation of fluorescently labelled silica materials. 1.0 g silica materials (such as spongy porous silica, hydrophobic spongy porous silica, MSNs, CALB/MSNs or Pd/MSNs) and 0.001 mmol 3-aminopropyltriethoxysilane (APTES) were added into 20 ml toluene. After stirring under a N_2 atmosphere at 60 ^oC for 2 h, the resultant solid particles were collected through filtration, washed four times with toluene and dried, resulting in amino-modified silica particles. 0.5 g amino-modified silica particles and 0.005 g fluorescein isothiocyanate isomer I (FITC-I) were added into 50 mL ethanol. The mixture was stirred overnight at room temperature in the dark. After filtration, the solid was washed five times with ethanol and dried under vacuum, yielding FITC-I-labelled silica. For the preparation of Rhodamine B-labelled silica, samples were prepared by a similar method but with water as the solvent.

4. Mechanical stability test

0.2 g SPs were filled into a quartz column (diameter of 0.7 cm and length of 10 cm) and were subjected to be fluidized with N_2 at a superficial velocity of 0.3 m s^{-1} for 24 h. Likewise, 0.5 g SPs were directly packed in a fixed-bed reactor and treated with flowing *n*-octane (flow rate: 6 mL h^{-1}) at 4 MPa N_2 (50 mL min^{-1}) for 48 h. After the treatment, the sample was collected for SEM observation and TG test.

5. Fluorescent permeability tests.

SPs were deposited on a glass slide. 30 μL Rhodamine B solution (2×10^{-5} M in a mixture of water and ethanol, V/V = 1:1) was gently dropped on the glass slide. The diffusion of Rhodamine B molecules into the interior of SPs was recorded by fluorescence microscopy at intervals.

6. Pd-CALB/SPs catalyzed cascade reactions.

Cascade of hydrogenation and kinetic resolution over Pd-CALB/SPs in batch system. 10 mg dodecane as an internal standard, 2 mL *n*-octane containing 0.1 M acetophenone and 0.4 M vinyl acetate were added to a 100 mL Teflon-lined steel autoclave. 30 mg Pd-CALB/MSNs, a physical mixture of 30 mg Pd/MSNs and 30 mg CALB/MSNs, 30 mg Pd-CALB@SPs, a physical mixture of 30 mg Pd/SPs and 30 mg CALB/SPs, 60 mg Pd-CALB/SPs (3.52-3.55 μmol Pd and 1.40-1.42 mg CLAB applied for all the reactions) were then added into the above solution, respectively. Before reaction, the autoclave was sealed and flushed with H_2 five times to remove the air. After being charged with a 2 MPa H_2 at room temperature, the autoclave was then heated to 50 °C while being stirred (600 rpm) for reaction to occur. The autoclave was allowed to cool to room temperature after reaction. The products were analyzed by GC at intervals and further confirmed with GC-MS. The catalyst was isolated from the reaction mixture via filtration, thoroughly washed with *n*-octane five times, and then dried at 50 °C under a vacuum, ready to be used in the next reaction cycle.

Sequential hydrogenation-kinetic resolution over Pd-CALB/SPs in continuous-flow system. In a typical reaction, 0.85 g Pd-CALB/SPs (0.050 mmol Pd; 19.8 mg CALB) and 0.2 g CALB/SPs (9.3 mg CALB) were mixed with quartz sand (120-160

mesh) to pack in a fixed-bed, at bottom and top of which were filled with quartz sand (40-60 mesh). Before reaction, the catalyst was flushed with H_2 at 2 MPa to remove air. Subsequently, a solution of ketone (0.1 M), vinyl acetate (0.4 M) and dodecane (internal standard) in *n*-octane as mobile phase was pumped through the inlet of the column reactor at a given flow rate and was allowed to pass through the column reactor whose temperature was kept at 50 °C. The outflow of the column reactor was sampled for GC analysis at regular intervals. The product was further confirmed with GC-MS.

Cascade reaction for the synthesis of chiral 1-phenylethanol acetate over H-beta-CALB/SPs in continuous-flow system. In a typical reaction, 1 g H-beta-CALB/SPs (CALB loading, 23.3 mg g^{-1}) was packed in a fixed-bed reactor. A solution of 1-phenylethanol (0.1 M) and vinyl acetate (0.4 M) in *n*-octane as mobile phase was pumped through the inlet of the column reactor at a given flow rate and was allowed to pass through the column reactor whose temperature was kept at 45 °C. The outflow of the column reactor was sampled for GC analysis at regular intervals. The product was further confirmed with GC-MS.

Sequential amine racemization-kinetic resolution over Pd-CALB/SPs in the continuous-flow system. In a typical reaction, 0.85 g Pd-CALB/SPs mixed with a small amount of quartz sand (120-160 mesh) were packed in a fixed-bed, at bottom and top of which were filled with quartz sand (40-60 mesh). Before reaction, the catalyst was flushed with 5% H_2/ 95% N_2 at 1 MPa (10 mL min^{-1}) to remove air. Subsequently, a solution of amine (0.1 M), ethyl methoxyacetate (0.2 M) and *n*-hexadecane (internal standard) in toluene as mobile phase was pumped through the inlet of the column reactor at a given flow rate (flow rate at 1.2 mL h^{-1} within first 190 h, flow rate at 1.0 mL h^{-1} from 190 to 290 h, flow rate at 0.72 mL h^{-1} from 290 to 390 h and 0.6 mL h^{-1} after 500 h.) and was allowed to pass through the column reactor whose temperature was kept at 70 °C. After running for 500 h, about 466 mL of the amine solution was used. The outflow of the column reactor was sampled for GC analysis at regular intervals. The product was further confirmed with GC-MS.

Supplementary Mass Spectrometry Data

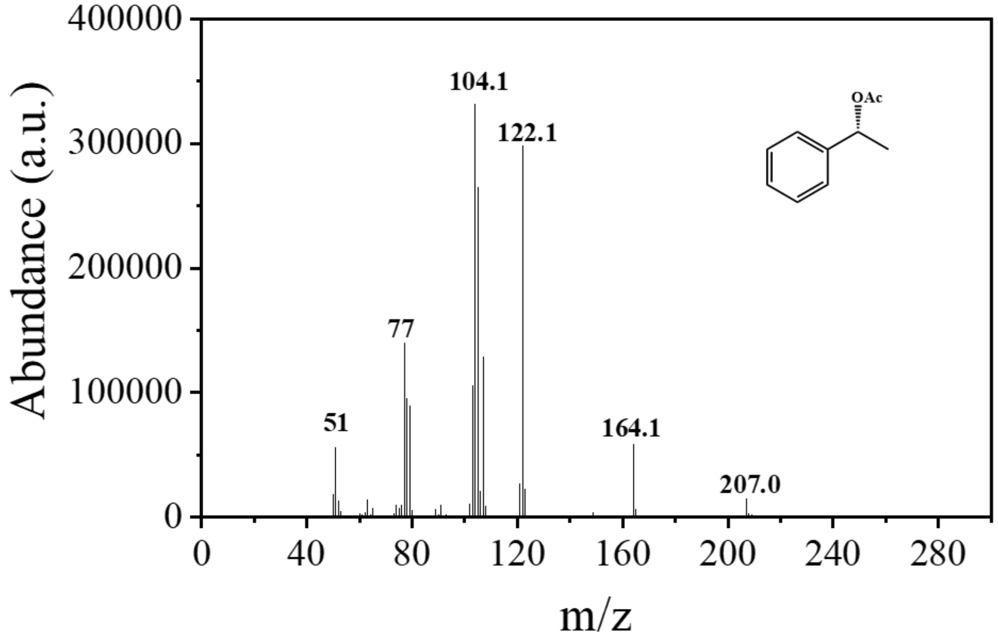

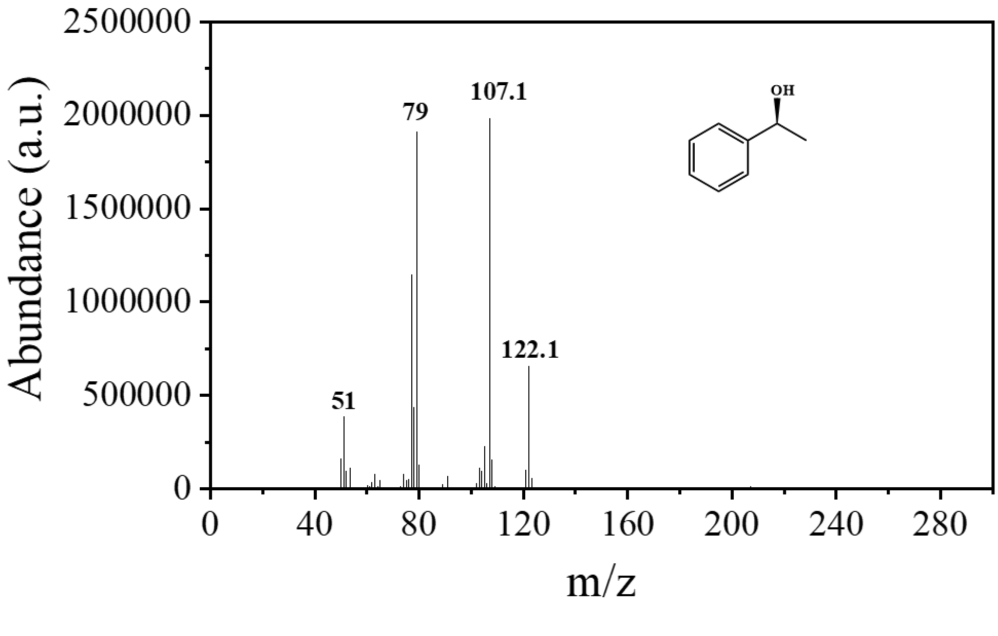

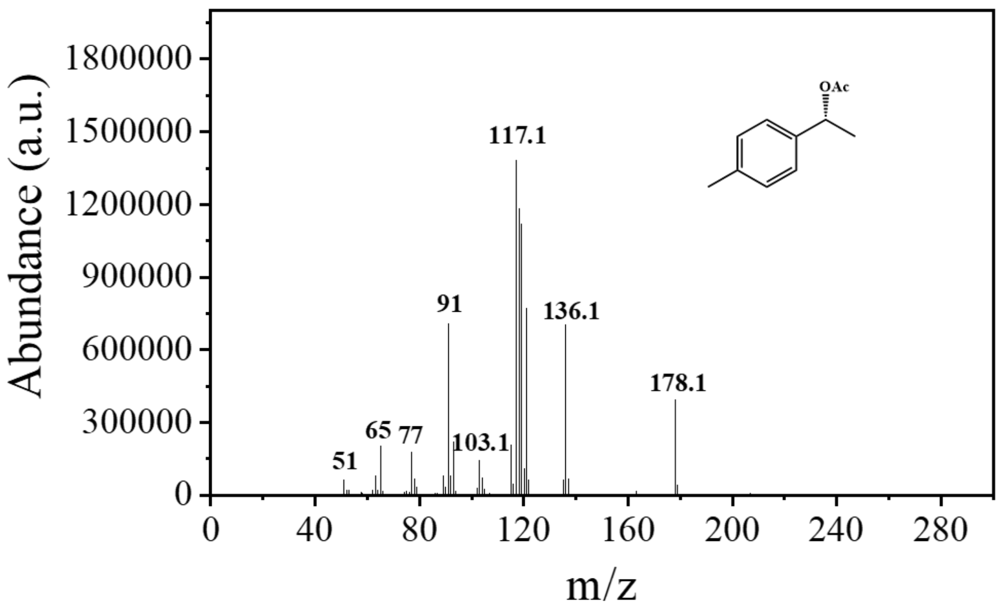

Abundance (a.u.)
m/z
0
300000
600000
900000
1200000
1500000
1800000
0
40
80
120
160
200
240
280
51
65
77
91
103.1
117.1
136.1
178.1
OAc

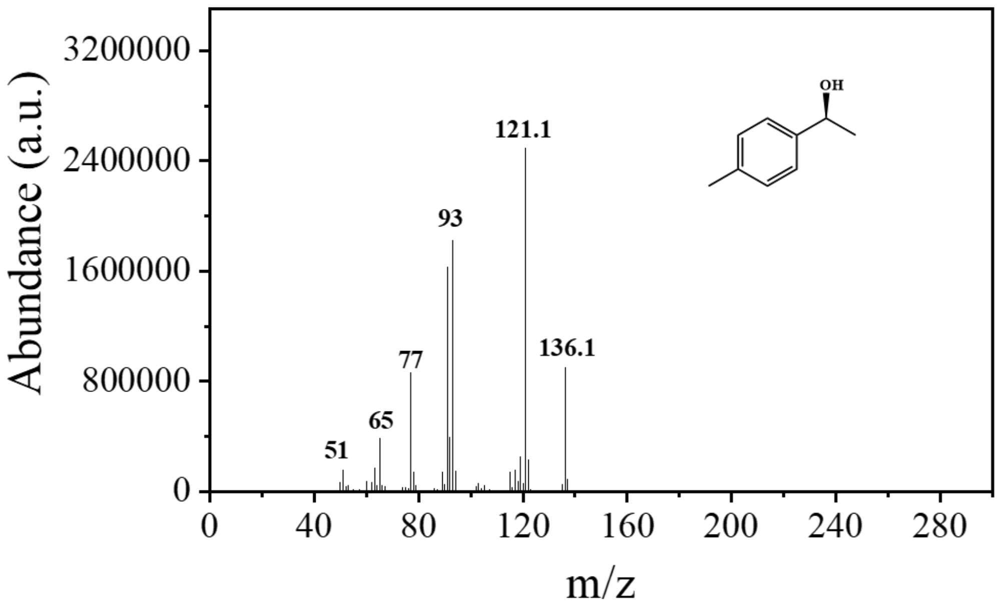

Abundance (a.u.)
m/z
0
800000
1600000
2400000
3200000
0
40
80
120
160
200
240
280
51
65
77
93
121.1
136.1
OH

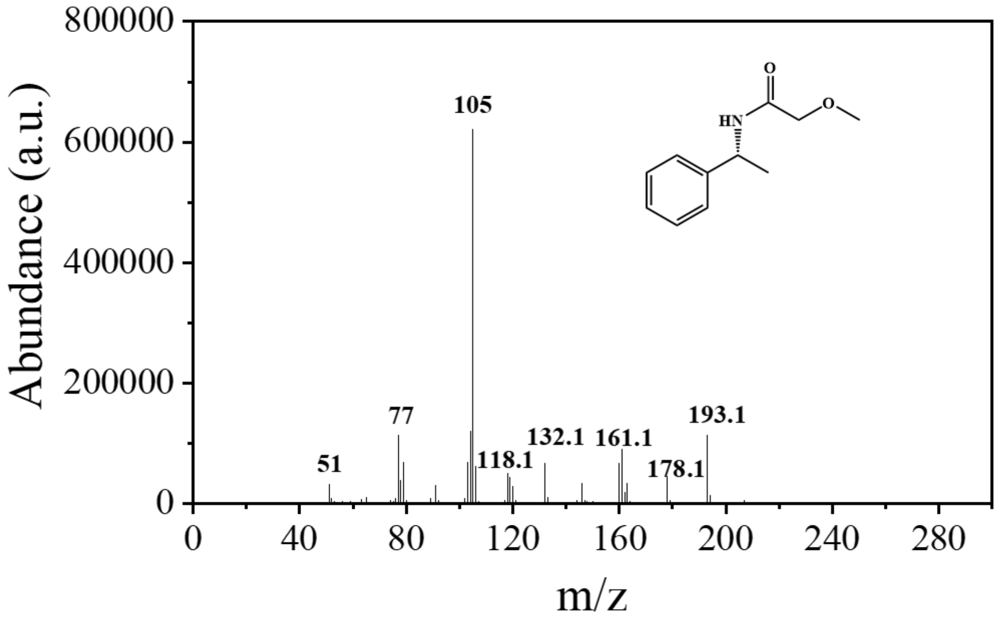

800000
600000
400000
200000
0
Abundance (a.u.)
105
77
51
118.1
132.1
161.1
178.1
193.1
0
40
80
120
160
200
240
280
m/z
O
HN
O

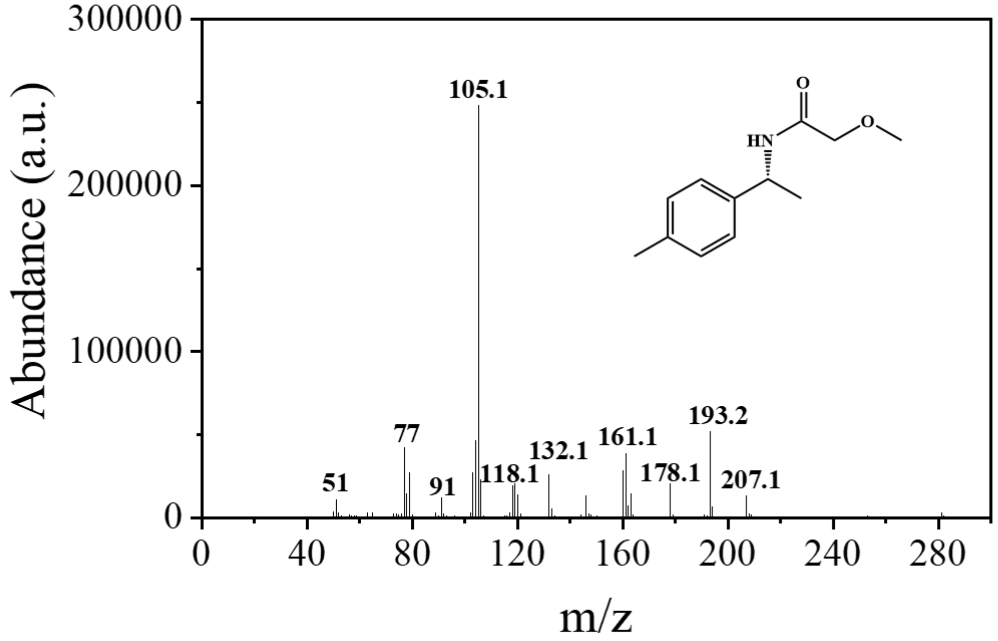

300000
200000
100000
0
Abundance (a.u.)
105.1
77
51
91
118.1
132.1
161.1
178.1
193.2
207.1
0
40
80
120
160
200
240
280
m/z
O
HN
O

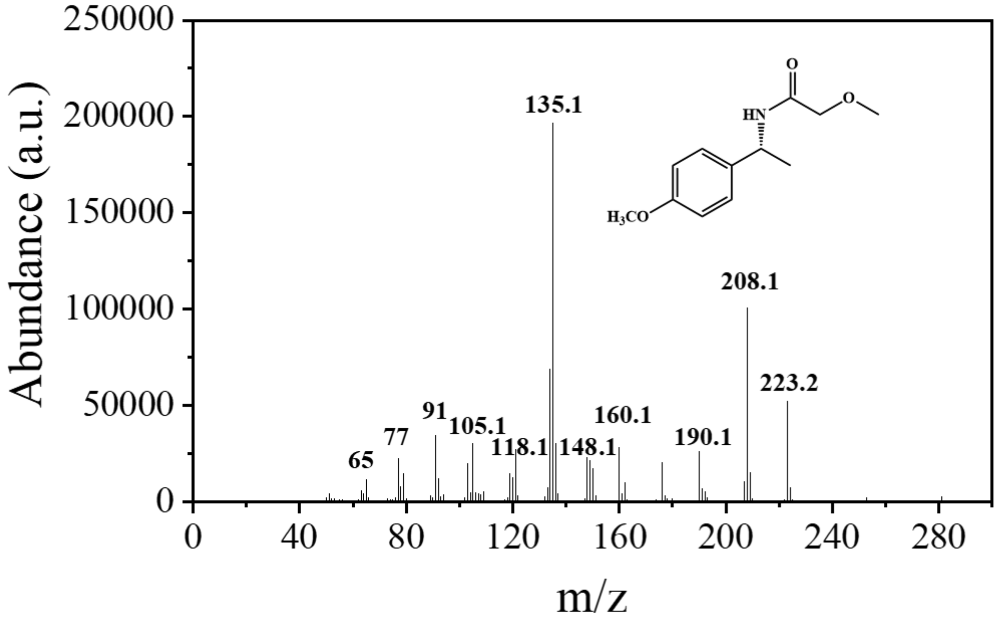

Abundance (a.u.)
m/z
135.1
208.1
223.2
160.1
190.1
148.1
118.1
105.1
91
77
65
HN
O
O
H3CO

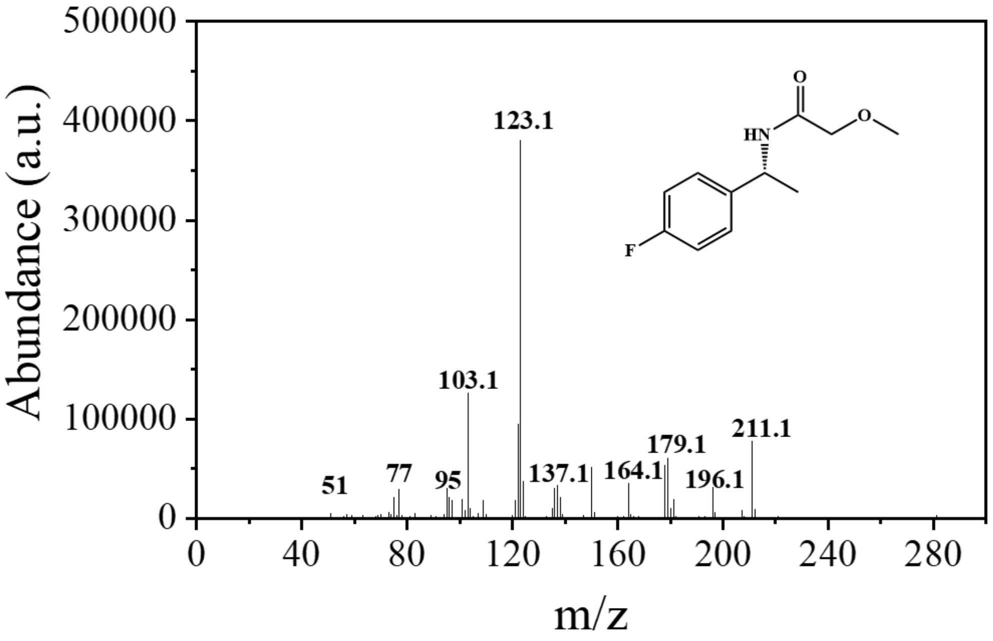

Abundance (a.u.)
m/z
123.1
103.1
211.1
179.1
164.1
196.1
137.1
95
77
51
HN
O
O
F

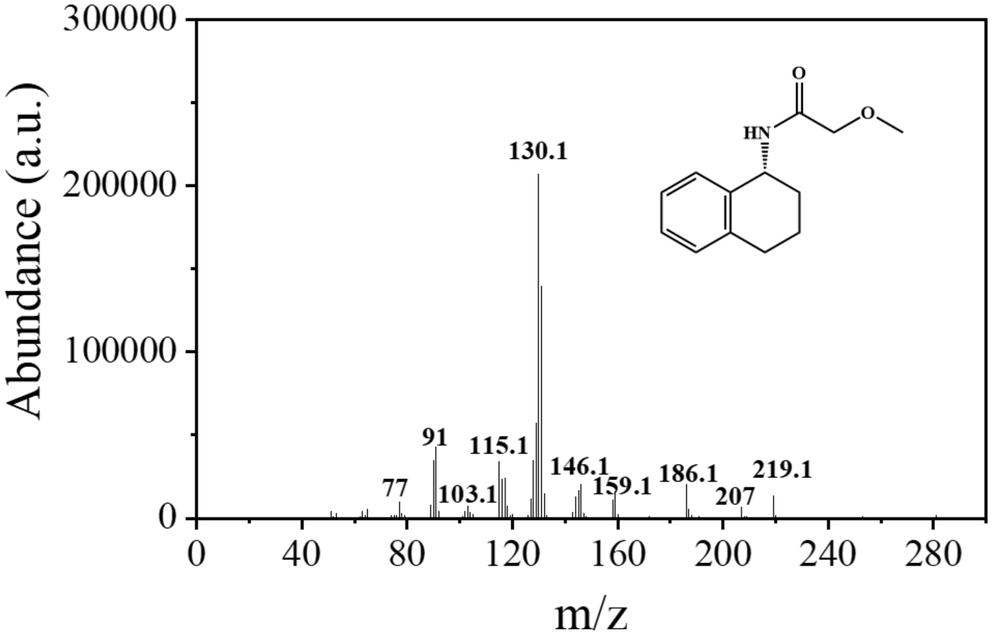
300000
200000
100000
0
Abundance (a.u.)
0
40
80
120
160
200
240
280
m/z
130.1
91
115.1
77
103.1
146.1
159.1
186.1
207
219.1
O
HN
O

Supplementary References

1. J. Sun, H. Zhang, R. Tian, D. Ma, X. Bao, D. S. Su, and H. Zou. Ultrafast enzyme immobilization over large-pore nanoscale mesoporous silica particles. *Chem. Commun*. **12,** 1322−1324 (2006).

2. Shen, D. K. et al. Biphase stratification approach to three-dimensional dendritic biodegradable mesoporous silica nanospheres. *Nano Lett*. **14**, 923−932, (2014).

3. Shen, D. K. et al. Ultradispersed Palladium Nanoparticles in Three-Dimensional Dendritic Mesoporous Silica Nanospheres: Toward Active and Stable Heterogeneous Catalysts. ACS Appl. *Mater. Interfaces* **7**, 17450−17459 (2015).

4. Luo, Z. et al. From Aggregation-Induced Emission of Au(I)–Thiolate Complexes to Ultrabright Au(0)@Au(I)–Thiolate Core–Shell Nanoclusters. *J. Am. Chem. Soc*. **134**, 16662−16670 (2012).
